# Oncolytic Adenovirus: Prospects for Cancer Immunotherapy

**DOI:** 10.3389/fmicb.2021.707290

**Published:** 2021-07-21

**Authors:** Yaqi Zhao, Zheming Liu, Lan Li, Jie Wu, Huibo Zhang, Haohan Zhang, Tianyu Lei, Bin Xu

**Affiliations:** Cancer Center, Renmin Hospital of Wuhan University, Wuhan, China

**Keywords:** oncolytic adenovirus, immunotherapy, genomic modification, cancer vaccine, immune checkpoint inhibitor

## Abstract

Immunotherapy has moved to the forefront of modern oncologic treatment in the past few decades. Various forms of immunotherapy currently are emerging, including oncolytic viruses. In this therapy, viruses are engineered to selectively propagate in tumor cells and reduce toxicity for non-neoplastic tissues. Adenovirus is one of the most frequently employed oncolytic viruses because of its capacity in tumor cell lysis and immune response stimulation. Upregulation of immunostimulatory signals induced by oncolytic adenoviruses (OAds) might significantly remove local immune suppression and amplify antitumor immune responses. Existing genetic engineering technology allows us to design OAds with increasingly better tumor tropism, selectivity, and antitumor efficacy. Several promising strategies to modify the genome of OAds have been applied: capsid modifications, small deletions in the pivotal viral genes, insertion of tumor-specific promoters, and addition of immunostimulatory transgenes. OAds armed with tumor-associated antigen (TAA) transgenes as cancer vaccines provide additional therapeutic strategies to trigger tumor-specific immunity. Furthermore, the combination of OAds and immune checkpoint inhibitors (ICIs) increases clinical benefit as evidence shown in completed and ongoing clinical trials, especially in the combination of OAds with antiprogrammed death 1/programed death ligand 1 (PD-1/PD-L1) therapy. Despite remarkable antitumor potency, oncolytic adenovirus immunotherapy is confronted with tough challenges such as antiviral immune response and obstruction of tumor microenvironment (TME). In this review, we focus on genomic modification strategies of oncolytic adenoviruses and applications of OAds in cancer immunotherapy.

## Introduction

With the global population aging, cancer has become a leading cause of death and a major barrier to improving quality of life in most countries (Sung et al., 2021). As the estimation of GLOBOCAN in 2020, almost 19 million new cases and 10 million cancer deaths occurred worldwide (Sung et al., 2021). Conventional chemotherapy, surgery, and radiotherapy for improving prognosis of the majority of cancer patients still have limitations. Accumulating evidence proves that tumor development, progression, and metastasis are closely associated with a dysfunctional immune system, also known as immunosuppression (Kobayashi et al., 2020). Immunotherapy is one of the most promising oncologic treatments that function through stimulating the immune system to attack tumor cells, thereby relieving tumor-induced immunosuppression (O’Donnell et al., 2019). In the early stage of immunotherapy, the focus was on regulating the functions of immune cells using immunostimulatory cytokines such as interferon-α (IFN-α) (Shehata et al., 2000) and interleukin-2 (IL-2) (Sim and Radvanyi, 2014) to enhance the antitumor immune response. Various forms of cancer treatments aimed at activating the immune system have been proposed currently, including immune checkpoint inhibitors (ICIs), chimeric antigen receptor (CAR) T cells, cancer vaccines, and oncolytic viruses (Zhao et al., 2019). Immunotherapies have shown increased overall survival (OS) and progression-free survival (PFS) of patients compared with standard chemotherapies. However, only a minority of cancer patients can benefit from immunotherapy due to immune-related toxicities (Kennedy and Salama, 2020) and lack of immunogenic signals to promote immune surveillance (Hegde and Chen, 2020). Thus, it is vital to develop a novel immunotherapeutic strategy with higher efficacy of activating the immune system and with less adverse events (AEs).

Oncolytic viruses (OVs) are naturally occurring or engineered viruses that selectively propagate in tumor cells while minimizing disruptions to normal ones (Cao et al., 2020). OVs can directly induce infection and lysis of malignant cells as well as being utilized as a vaccine to trigger tumor-specific immunity. Simultaneously, OVs may express therapeutic transgenes for amplifying antitumor immune responses, such as cytokines, tumor antigens, or checkpoint inhibitors (Evgin et al., 2020). In addition, recent studies prove that OVs combined with immune checkpoint inhibitors (ICIs) is an ideal strategy for cancer therapy (Samson et al., 2018). Compared with tumors with a scanty infiltration of immune cells, “hot” tumors which have an abundance of infiltrating immune cells in the tumor microenvironment (TME) are more likely to respond to ICIs (Gujar et al., 2018b). The immune responses induced by OVs turn tumors “hot” via promoting the cytokine secretion and recruitment of CD8^+^ T cells, which increases efficacy of ICIs (Gujar et al., 2018a).

Currently, there are numerous OVs under preclinical or clinical investigations, including but not limited to adenoviruses (Lang et al., 2018), herpes viruses (Andtbacka et al., 2015), reoviruses (Mahalingam et al., 2020), and poxviruses (Chaurasiya et al., 2020). Remarkably, talimogene laherparepvec (T-VEC), based on oncolytic herpes virus, was the first OV approved by the Food and Drug Administration (FDA) for melanoma treatment in 2015 (Masoud et al., 2019). Another commonly employed and well-studied OVs is oncolytic adenovirus (OAd) because of its powerful capacity in oncolysis and immune response stimulation (Robert-Guroff, 2007). In this review, we will concentrate on the main genomic modification strategies of OAds and applications of OAds as cancer vaccines or as combinatorial partners for ICIs. We enumerate the OAds that have shown promising efficacy and safety in preclinical or clinical trials over the last 20 years. Additionally, we highlight some challenges faced by OAds and their possible solutions.

## Structure and Function of Oncolytic Adenovirus

Adenovirus (Ad) is a non-enveloped, double-stranded linear DNA virus with icosahedral capsid that mainly consists of hexon, penton, and fiber proteins (Chen and Lee, 2014). The penton protein anchors the protruding fiber to 12 vertices of icosahedral virion (Arnberg, 2012). The viral genome encodes early (E1A, E1B, E2, E3, and E4) and late transcription units (L1, L2, L3, L4, and L5) that produce multiple mRNA and proteins during the replication and aggregation of Ad (McConnell and Imperiale, 2004). At least 103 adenovirus types have been identified up to 2019 (Table 1), they are divided into seven species (A–G) based on oncogenicity, hemagglutination properties, DNA homology, and genome organization (Gao et al., 2020a). Adenoviruses are tightly associated with human disease worldwide, particularly types 1, 2, 3, 4, 5, 7, 8, 21, 37, 41, 53, 54, 56, and 64 (Lion, 2014; Jonas et al., 2020). Ads can infect the respiratory tract (species B, C, and E), the gastrointestinal tract (species F and G), and ocular surface (species B, D, and E) (Radke and Cook, 2018). Of note, epidemic keratoconjunctivitis (EKC) is a severe ocular surface infection caused by adenovirus species D types 8, 37, 53, 54, 56, and 64 (Jonas et al., 2020). Outbreaks of EKC occur worldwide but are more frequent and larger in Southeast Asia, imposing a tremendous health and socioeconomic burden (Ismail et al., 2019). Epidemiological data indicated that most primary Ad infections occur during the first 5 years of life (Lion, 2014). Due to the high prevalence of Ad, over half of individuals present preexisting immunity to Ad, especially adenovirus type 5 (Ad5) (Yu et al., 2012).

**TABLE 1:** Overview of the identified receptors of adenovirus species and serotypes.

Species	Types	Receptor(s)
A	12, 18, 31, 61	CXADR
B	3, 7, 11, 14, 16, 21, 34, 35, 50, 55, 66, 68, 76–79	CD46, desmoglein-2, CD80/86
C	1, 2, 5, 6, 57, 89	CXADR, VCAM-1, HSPG, MHC-Iα2, scavenger receptor
D	8–10, 13, 15, 17, 19, 20, 22–30, 32, 33, 36–39, 42–49, 51, 53, 54, 56, 58–60, 62–65, 67, 69–75, 80–88, 90–103	Sialic acid, CD46, CXADR
E	4	CXADR
F	40, 41	CXADR
G	52	CXADR, polysialic acid

*CXADR, coxsackievirus and adenovirus receptor; VCAM-1, vascular cell adhesion molecule-1; HSPG, heparin sulfate proteoglycans; MHC1-a2, major histocompatibility complex-I α2. The data of adenovirus species and serotypes are from HAdV Working Group (http://hadvwg.gmu.edu/).*

**FIGURE 1:**
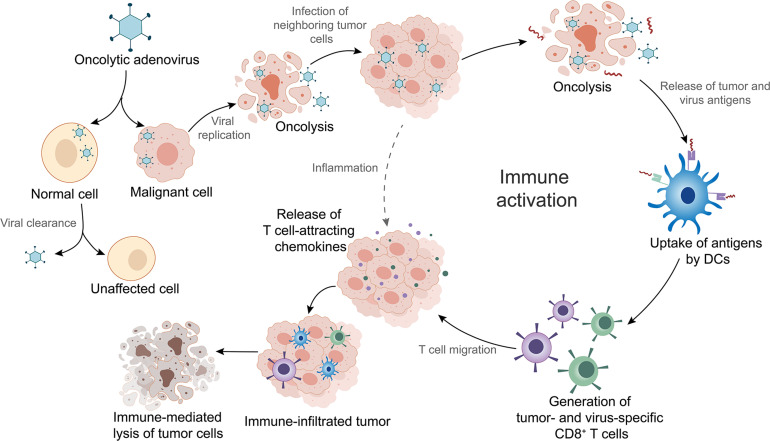
Mechanism of oncolytic adenoviruses (OAds) in cancer immunotherapy. OAds selectively enter into malignant cells while being cleaned up by normal cells. Subsequent viral replication leads to tumor cell lysis and release of virus- and tumor-specific antigens. These antigens are picked up by dendritic cells (DCs) and presented to T cells, which initiate local antitumor immune activation. Activated T cells migrate into the tumor tissues, where T-cell attracting chemokines recruit more immune cells, facilitating tumor immune infiltration and enhancement of immunotherapy efficacy. Furthermore, infection by OAds can also induce inflammation that contributes to immune infiltration.

OAd is a therapeutic agent that infects tumor cells via the interaction of adenoviral fiber knob to receptors on the surface of the cells (Baker et al., 2019). Different types of OAds enter into tumor cells through different receptors. For instance, Ad5 binds predominantly to the coxsackievirus and adenovirus receptor (CAR/CXADR) (Lopez-Gordo et al., 2017), whereas adenovirus type 3 (Ad3) has a high binding affinity for desmoglein-2 (Wang H. et al., 2011), CD46, CD80, or CD86 (Hall et al., 2009). OAds possess remarkable intratumoral amplification and oncolytic property. During infection, the adenoviral DNA genome is transported into the nucleus leading to initiation of E1A gene transcription (McConnell and Imperiale, 2004). The subsequent transcription of other early and late genes activated by E1A proteins leads to expression of viral proteins and production of progeny viruses (McConnell and Imperiale, 2004). Thousands of emerging viruses result in tumor cell lysis as well as in the release of tumor and virus antigens in the tumor microenvironment (TME) (Fusciello et al., 2019). The antigens are subsequently presented to T cells by dendritic cells (DCs), which is an important part of activating local antitumor immune response (Tuve et al., 2009; Figure 1). Additionally, OAds can be altered to obtain higher antitumor potency with lower toxicity through genomic modifications (Hemminki and Hemminki, 2016). In the following sections, we will introduce the mainstream genomic modification strategies of OAds in detail.

## Genomic Modifications of Oncolytic Adenovirus

Although wild-type adenoviruses preferentially infect tumor cells due to the defective viral sensing mechanism of most tumor cells (Xia et al., 2016), OAds can achieve better therapeutic results by rational design. Capsid modification was used to improve tumor tropism. For enhancing tumor selectivity, small deletions in the pivotal viral genes and insertion of tumor-specific promoters are being considered. Besides, OAds acquired high antitumor efficacy by adding immunostimulatory transgenes.

### Improving Tumor Tropism

Ad5 is the most commonly used backbone for conventional OAds design, whereas CXADR, the natural receptor of Ad5, is absent or low expressed in many cancer cells (Koodie et al., 2019). Unfortunately, low receptor expression can drastically hinder effective entry of OAds into tumor cells. To address this conundrum, adenoviruses can be redirected to other receptors by modifying the adenoviral fiber-knob of the capsid.

The first modification strategy was chimeric fibers. Replacement of the Ad5 fiber knob with Ad3 fiber knob (5/3 chimerism) has been proven to increase infectivity for cancer cells, while retaining the safety and replication competent of the parental Ad5 (Koodie et al., 2019). Notably, Ad5/3 might overcome neutralization of preexisting neutralizing antibodies in the blood through competitively binding to lymphocytes and erythrocytes (Zafar et al., 2020b). Chimeric 5/35 adenoviral vector (Ad5/35) also shows elevated cell entry and infection in gastric cancer cells (Wang et al., 2013), hepatocellular carcinoma (Chen et al., 2011), and bladder cancer cells (Do et al., 2018). Moreover, Ad5/37 and Ad3/11p chimeric viruses were generated to enhance transduction rates in OAds, which have been used in multiple trials (Machiels et al., 2019; Gao et al., 2020b).

The second strategy is an incorporation of arginine-glycine-aspartic acid (RGD) peptides in the fiber knobs of Ad5, which allows the virus to enter cells using α_*v*_β_3_ or α_*v*_β_5_ integrins (Fueyo et al., 2003). A phase I clinical study of RGD modified oncolytic adenoviruses has shown promising results that 20% of glioma patients achieved antiglioma immune responses with long-term survival (Lang et al., 2018).

As it is difficult for Ad5 to access tumor cells, researchers have constructed fully serotype 3 OAds instead. In mice, Ad3 was more immunogenic and induced higher amount of cytokines but less liver damage than Ad5 and Ad5/3 (Hemminki et al., 2012). In 15 patients with chemotherapy refractory cancer, signs of antitumor activity were seen in 73% patients after treating with engineered Ad3 (Hemminki et al., 2012). Zafar et al. constructed TILT-234, an Ad3-based oncolytic adenovirus, to help recruit DCs and stimulate antigen presentation, which might facilitate DC therapy in patients with prostate cancer (Zafar et al., 2020a).

### Enhancing Tumor Selectivity

**FIGURE 2:**
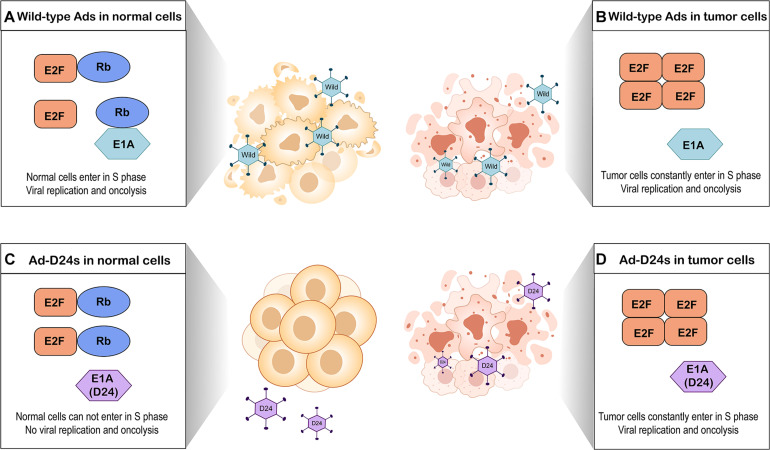
Tumor selectivity of oncolytic adenoviruses with E1A gene 24-base pair deletion (Ad-D24s) and oncolytic adenoviruses without genomic modification (wild-type Ads). **(A)** Wild-type Ads infect normal cells. E1A protein of adenovirus interferes with Rb protein by binding it, leading to E2F release and accumulation. Free E2F allows the normal cells to enter into S phase of the cell cycle, which results in viral replication and oncolysis. **(B)** Wild-type Ads infect tumor cells. Due to the defective Rb pathway, the accumulation of free E2F allows tumor cells to constantly enter in S-phase. Wild-type Ads can replicate in the tumor cells and lyse them. **(C)** Ad-D24s infect normal cells but there is no viral replication and oncolysis. Mutated E1A protein cannot bind to Rb protein; therefore, E2F is still inactivated by Rb protein. The normal cells are unable to enter into S phase. **(D)** Tumor cells constantly enter in S-phase because of the defective Rb pathway; Ad-D24s can also lyse the tumor cells while generating viral progeny.

A major restriction of natural Ads is the lack of the ability to selectively target tumor cells (Lee et al., 2020). To enhance tumor selectivity and to reduce unwanted AEs, various modifications have been proposed. The E1B region of the adenoviral genome encodes a 55-kilodalton protein (E1B-55K) that binds and inactivates the cellular tumor suppressor protein p53 (Bischoff et al., 1996). An OAd with a E1B-55K gene deletion which replicated only in p53-deficient tumor cells but not cells with functional p53 was first described in 1996 (Bischoff et al., 1996). Subsequently, several studies have reported other modifications such as E1B-19K gene deletion (E1B-19 kD) (Liu et al., 2004), E3 gene deletion (Wang et al., 2003), and E1A gene 24-base pair deletion (Heise et al., 2000). Among them, E1A gene 24-base pair deletion is widely employed to modify OAds. E1A gene conserved region 2 (CR2) encodes E1A protein sequence which can bind to and inactivate retinoblastoma (Rb) proteins (Heise et al., 2000). The Rb protein is considered to be a tumor suppressor, which functions through binding to E2F and thus inhibiting cell cycle progression (Kitajima et al., 2020). E2F acts as a transcriptional activator to regulate expression of key genes that enable the quiescent cells to enter into S-phase (Kent and Leone, 2019). To prevent normal cells from entering in S-phase, OAds with E1A gene 24-base pair deletion express mutated E1A proteins which cannot interfere with Rb proteins. This process blocks viral replication in quiescent normal tissues (Lang et al., 2018). In contrast, OAds with this deletion replicate in most tumor cells because the defective Rb pathway in tumor cells prevents cell cycle arrest and permits tumor cells to constantly enter in S-phase (Marshall et al., 2019; Figure 2).

However, relying solely on viral genes deletion is insufficient for resolution of unwanted tissue damage (Pesonen et al., 2012). Another approach to control viral replication is inserting tumor-specific promoters. For instance, inserting human telomerase reverse transcriptase (hTERT) promoter into viral gene enables OAds to replicate only in cells with high-telomerase activity—a hallmark of cancer (Hemminki et al., 2012). Similarly, other tumor-specific promoters have also been adopted including the E2F-1 promoter (Hemminki et al., 2015), p53 promoter (Wang et al., 2008), cyclo-oxygenase 2 (cox2) promoter (Bauerschmitz et al., 2006), and the α-fetoprotein (AFP) promoter (Kim et al., 2002).

### Increasing Antitumor Efficacy

The growing recognition that immune activation induced by OAds is an essential component of cancer treatment has led researchers to construct potent vectors for immune factors through adding immunostimulatory transgenes into the adenoviral genome. This allows these immunostimulatory factors to accumulate at tumor sites, thereby increasing antitumor efficacy and decreasing systemic adverse reaction. Several common immune factors carried by OAds are described below.

#### GM-CSF

Granulocyte-macrophage colony-stimulating factor (GM-CSF) is best known for its critical role in immune modulation and hematopoiesis (Hong, 2016). Some studies have suggested GM-CSF also promotes tumor cell proliferation and migration (Kowanetz et al., 2010; Curran et al., 2011). However, increased attention is now focused on the activation of long-lasting antitumor immune response through stimulating DC maturation and monocyte/macrophage activity (Hong, 2016). Adding a GM-CSF transgene into the adenoviral genome promoted the recruitment and activation of DCs that further enhance tumor antigen presentation to T cells (Kanerva et al., 2013). CG0070 is a conditionally replicating OAd engineered by inserting the E2F-1 promotor and the human GM-CSF gene (Ramesh et al., 2006). A phase I trial of CG0070 recruited 35 patients with non-muscle-invasive bladder cancer who had failed to respond to bacillus Calmette-Guerin (BCG) treatment (Burke et al., 2012). 48.6% of the patients achieved complete responses with a median duration of 10.4 months. The complete response rate increased to 63.6% in the patients received multiple intravesical infusion of CG0070 (Burke et al., 2012). Subsequently, the efficacy and acceptable safety of CG0070 has been reported in a single-arm phase II trial (NCT02365818). Almost half of patients achieved complete response at 6 months with an acceptable incidence of treatment-related AEs (Packiam et al., 2018). ONCOS-102 (Ad5/3-D24-GMCSF), another OAd coding for GM-CSF, effectively eradicated human melanoma cells and induced complete tumor regression in the xenograft model (Bramante et al., 2015). The GM-CSF expressed by viruses stimulated the differentiation of monocytes to macrophages (Bramante et al., 2015). Due to superior performance in preclinical experiments, ONCOS-102 was tested in a phase I clinical trial (NCT01598129) which enrolled 12 patients with refractory solid tumors (Ranki et al., 2016). ONCOS-102 was safe and well tolerated in all patients and median OS was 9.3 months. Eleven patients showed a short-term increase in systemic proinflammatory cytokines and tumor infiltrating lymphocytes (TILs), especially CD8^+^ T cells. Two patients with the best overall survival showed the most prominent infiltration of CD8^+^ T cells to tumors as well as systemic induction of tumor-specific CD8^+^ T cells. Importantly, the upregulated expression of PD-L1 in the post-treatment of mesothelioma patients further highlighted that ONCOS-102 can act as an immunostimulatory agent to facilitate immune checkpoint blockade (Ranki et al., 2016). More details of these combinatory therapies will be described in later chapters.

#### IL-12

Interleukin-12 (IL-12) is a proinflammatory cytokine that initiates antitumor immune responses by promoting the generation of tumor-specific cytotoxic T lymphocytes (CTLs) and activating natural killer (NK) cells and T cells (Colombo and Trinchieri, 2002). Although local IL-12 therapy is consistently considered to be a potent approach to overcome tumor-induced immune suppression (Nguyen et al., 2020), unfortunately, systemic accumulation of IL-12 results in a potential lethal inflammatory syndrome (Leonard et al., 1997). Therefore, delivering it by OAds seems critical for maximizing the density of IL-12 that reaches the tumors and alleviating the toxicity.

Wang P. et al. (2017) constructed an OAd (Ad-TD-nsIL-12) with triple gene deletions (E1ACR2, E1B19K, and E3gp19K) to deliver non-secreting IL-12 (nsIL-12) into tumor cells. This modified IL-12 without the N-terminal signal peptide was released at considerably higher levels in Syrian hamster pancreatic ductal adenocarcinoma cells than normal cells so that the dissemination of IL-12 was limited to the local TME (Wang P. et al., 2017). Barrett et al. designed a replication-incompetent adenoviral vector containing murine IL-12 gene (Ad-RTS-mIL-12) and regulated the transcription of the IL-12 gene by the RheoSwitch Therapeutic System (RTS), a gene expression control switch platform (Barrett et al., 2018). Under the control of RTS, the IL-12 gene was exclusively transcribed in the local tumor tissues initiated by RheoSwitch activator ligand, veledimex (Komita et al., 2009). Localized controlled production of IL-12 which was induced by intratumorally injecting Ad-RTS-mIL-12 and orally administering veledimex correlated with an increase in TILs and prolonged survival in an orthotopic glioma model (Barrett et al., 2018). Ad-RTS-hIL-12 encoding human IL-12 p70 transgene is another OAd transcriptionally regulated by veledimex (Chiocca et al., 2019). Chiocca et al. (2019) reported a phase 1 dose-escalation study (NCT02026271) to evaluate safety and tolerability of a fixed intratumoral Ad-RTS-hIL-12 dose with variable veledimex doses in 31 patients with recurrent high-grade glioma. After the administration of Ad-RTS-hIL-12 and veledimex, sustained increased IFN-γ concentration were found in the tumor microenvironment. In addition, the concentrations of IL-12 and IFN-γ in the peripheral blood showed positive correlations with veledimex dose. Five patients with suspected recurrence after Ad-RTS-hIL-12 injection showed evidence of increased TILs producing IFN-γ and PD-1, suggesting that they were due to pseudoprogression and hIL-12 can mediate immunological effects (Chiocca et al., 2019). Unfortunately, the safety of Ad-RTS-hIL-12 cannot be evaluated because the treatment is delivered during neurosurgical resection and the AEs caused by surgery are difficult to exclude.

#### CD40L and 4-1BBL

As multiple cells in the tumor stroma, such as the infiltrating immune cells, express CD40 and 4-1BB, immunotherapies targeting CD40 and 4-1BB have gained growing interest (Eriksson et al., 2017a). CD40 showed a tremendous ability to drive macrophage differentiation and stimulate T-helper (Th) 1 immunity by promoting DC maturation (Eriksson et al., 2017b). Previous studies reported that binding of CD40 to its natural ligand, CD40L, induced the apoptosis of cancer cells (Korniluk et al., 2014). 4-1BB is expressed on activated T and NK cells acting as an inducible costimulatory receptor to interact with its major biological ligand, 4-1BBL, which present on activated professional antigen presenting cells (APCs) including DCs, macrophages, and B cells (Alderson et al., 1994; Chin et al., 2018). The interaction between 4-1BB and 4-1BBL elicited the proliferation of activated thymocytes and splenic T cells (Goodwin et al., 1993). Otherwise, agonistic monoclonal antibodies targeting 4-1BB enhanced tumor clearance and durable antitumor immune responses in induced and spontaneous tumor models (Chester et al., 2018). LOAd703 is an adenovirus armed with trimerized CD40L and 4-1BBL to stimulate CD40 and 4-1BB pathways that activated antitumor effects (Eriksson et al., 2017a). As pancreatic cancer with a high level of M2 macrophages has responded to anti-CD40 agonist therapy, LOAd703 was tested in pancreatic cell lines. The efficacy of this virus in killing pancreatic cancer cells by oncolysis was even better than gemcitabine, the standard adjuvant chemotherapy of pancreatic cancer. Furthermore, human Panc01 cells were injected subcutaneously in immunodeficient mice to establish xenograft models. LOAd703 efficiently reduced established tumors after six peritumoral injections and achieved additional effects in combination with gemcitabine (Eriksson et al., 2017a). Recently, Wenthe et al. (2020) selected six human multiple myeloma (MM) cell lines to evaluate the feasibility and efficacy of LOAd703 for MM treatment. LOAd703 could infect and lyse MM cells at even low virus to cell ratio. Notably, LOAd703 infection induced the downregulation of markers related to MM progression (ICAM-1, CD70, CXCL10, CCL2, and sIL-2Rα) while the apoptosis receptor Fas was upregulated. In the coculture systems of immune and MM cells, LOAd703 obviously promoted activation of CTLs with increased expression of CD69, CD107a, and IFN-γ (Wenthe et al., 2020).

#### IL-2 and TNF-α

IL-2 is the predominant factor responsible for the T-cell proliferation and differentiation, which has been approved to treat metastatic melanoma and renal cancer (Rosenberg, 2014). Tumor necrosis factor-α (TNF-α), a multifunctional cytotoxic molecule that cannot only induce tumor cell apoptosis and necrosis but can also stimulate release of other cytokines and recruit immune cells (Hirvinen et al., 2015). Havunen et al. (2017) constructed an OAd based on the backbone of Ad5/3-E2F-d24 carrying human IL-2 and TNF-α (TILT-123 or Ad5/3-E2F-d24-TNFa-IRES-hIL2) and used them to improve adoptive TIL transfer. All tumors in immunocompetent animal models were cured by combining TILT-123 with TILs (Havunen et al., 2017). When TILT-123 was injected into mouse melanoma models concomitantly receiving anti-PD-1 therapy, all the tumors regressed completely and 100% of the mice remained alive by day 90 (Cervera-Carrascon et al., 2018). TILT-123 has also been regarded as an attractive alternative agent to host lymphodepletion in solid tumor adoptive T-cell therapy (ACT) due to its acceptable toxicity and high anti-tumor efficacy (Santos et al., 2018). Furthermore, treatment with TILT-123 could reconfigure the tumor microenvironment to accommodate heightened anti-tumor TIL reactivity in an *ex vivo* tumor model derived from ovarian cancer (OVCA) patient samples (Santos et al., 2020). Increased proinflammatory signals (IFN-γ, CXCL10, TNF-α, and IL-2) and concomitant activation of CD4^+^ and CD8^+^ TILs were observed in the ovarian tumor cells infected by TILT-123 (Santos et al., 2020). Given these encouraging findings in preclinical studies, TILT-123 is employed in an ongoing clinical trial (NCT04217473), where patients with advanced melanoma receive combined TILT-123 and TIL therapy.

#### OX40L

In addition to T-cell receptor (TCR)-mediated antigen-specific signal transduction, optimal activation of T cells requires antigen engagement with positive secondary signals provided by costimulatory molecules such as OX40 (CD134), a tumor necrosis factor receptor super family member (Hewitt et al., 2019). OX40 can promote T-cell survival, increase cytokine production, and enhance T-cell migration by interacting with its cognate ligand OX40L (Webb et al., 2016). Jiang et al. (2017) first reported Delta-24-RGDOX, an OAd-expressing OX40L, induced immunogenic cell death and superior tumor-specific activation of lymphocytes in syngeneic glioma mouse models. Since then, the virus was tested in immunocompetent mice with disseminated melanomas (Jiang et al., 2019). Localized treatment with Delta-24-RGDOX mediated tumor-specific T-cell expansion and migration, resulting in efficacious immune activation which is sufficient to elicit an abscopal antimelanoma effect, even in the brain (Jiang et al., 2019).

#### Dilemmas in Developing OAds Armed With Immunostimulators

Although high expression levels of immune effectors and substantial reductions in AEs have been observed in preclinical models, the development of OAds armed with immunostimulatory cytokines and chemokines is a tough task. The primary problem to be addressed is to develop adequate animal models that permit human adenovirus replication *in vivo* and simultaneously reflecting the host’s immune response. Immunodeficient mice bearing human tumor-derived xenografts are used in virtually all *in vivo* experiments of OAds since human Ads cannot be allowed to replicate in mouse cells (Machitani et al., 2016). However, the antitumor immune responses induced by viruses will not be completely reflected in immunodeficient animals and the therapeutic effects in patients cannot be accurately predicted. For these reasons, some OAds showed promising results in preclinical trials but turned out to be less successful in clinical trials. To fully assess the efficacy and safety of OAds carrying immunostimulatory molecules, more clinical trials and construction of human organoid models might constitute valuable solutions.

## Oncolytic Adenovirus as a Cancer Vaccine

Most tumors are poorly immunogenic and fail to elicit immune responses on their own, which may be due to their low mutational burden or elevated immunosuppression signals through activation of β-catenin pathway (Spranger et al., 2015; Maeng et al., 2018). In these cases, vaccines can provide the absent immunogenicity, enhancing antitumor capacity and blocking tumor growth, metastasis, and recurrence (Sarvizadeh et al., 2019). Cancer vaccines can target tumor-specific antigens (TSAs) or tumor-associated antigens (TAAs) expressed on tumor cells to trigger active immune response against tumors (Tran et al., 2019). Previous researches have uncovered a wide variety of cancer vaccine platforms, including peptide based, protein based, bacterial or viral based, and pulsed dendritic cells (Gatti-Mays et al., 2017). Intriguingly, therapeutic cancer vaccines based on adenovirus vectors have been extensively applied to amplify antitumor immune responses to transgenes expressed by the vectors. Table 2 lists clinical trials of OAd-based cancer vaccines.

**TABLE 2:** Clinical trials on OAd-based cancer vaccines.

OAd name	Transgene	Indication	Combination therapy	Clinical trial number	Phase
ETBX-011 (Ad5 [E1-, E2b-]-CEA(6D)	CEA	Colon cancer Lung cancer Breast cancer	–	NCT01147965	I/II
		Neoplasms Prostate cancer Lung cancer Breast cancer Colon cancer	ETBX-061 (Ad5 [E1-, E2b-]-MUC-1) ETBX-051 (Ad5 [E1-, E2b-]-brachyury)	NCT03384316	I
Ad5-PSA	PSA	Hormone refractory prostate cancer	–	NCT00583024	II
ETBX-071 (Ad5 [E1-, E2b-]-PSA)		Metastatic castration-resistant prostate cancer	ETBX-061 (Ad5 [E1-, E2b-]-MUC-1) ETBX-051 (Ad5 [E1-, E2b-]-brachyury)	NCT03481816	I
Ad-MAGEA3	MAGE-A3	Advanced/metastatic solid tumor	MG1-MAGEA3	NCT02285816	I/II
		Non-small cell lung cancer	MG1-MAGEA3, pembrolizumab	NCT02879760	I/II

### Ad5 [E1-, E2b-]-CEA(6D) or ETBX-011

Carcinoembryonic antigen (CEA), an oncofetal glycoprotein antigen involved in cell adhesion, is present in the intestine, liver, and pancreas of the fetus. Measurement of CEA has been recommended as a prognostic indicator in colorectal cancer as well as a surveillance tool for monitoring the tumor recurrence (Miksad and Meropol, 2018).

An advanced Ad5 vector gene delivery platform with deletions of E1, E2b, and E3 gene regions, known as Ad5 [E1-, E2b-], was constructed as a cancer vaccine regardless of the existence of pre-existing Ad5 immunity (Gabitzsch et al., 2015). Based on this platform, ETBX-011 (Ad5 [E1-, E2b-]-CEA(6D) was established to induce potent CEA-specific cell-mediated immunity (CMI) through inserting the modified CEA gene that encodes the highly immunogenic CAP1-6D peptide (Morse et al., 2013). CAP1-6D modification of CEA has been shown 100–1,000 times more efficient in enhancing the sensitization to CTLs, compared with the naïve CAP1 epitope (Zaremba et al., 1997). Multiple phase I/II trails are now under way or have been completed to evaluate the therapeutic efficacy of ETBX-011. Morse et al. recruited 32 patients with metastatic colorectal cancer into a dose-escalation trial (NCT01147965) (Morse et al., 2013). No dose-limiting toxicities (DLTs) and AEs that resulted in treatment discontinuation were observed in 32 patients, and the most common toxicity was a self-limited, injection site reaction. CEA-specific CMI responses were still observed in the setting of Ad5-specific immunity existing in 61.3% of patients. Importantly, 25 patients who were treated with ETBX-011 at least two times experienced a 12-month survival probability of 48% (Morse et al., 2013). In their extended evaluation on long-term overall survival, the 29-month overall survival probability of all 32 patients was 20% with a median OS of 11 months (Balint et al., 2015). Peripheral blood mononuclear cells (PBMC) samples from two patients were available for additional immune analyses, which detected activated CD4^+^ and CD8^+^ T cells in a postimmunization sample with high CMI activity. Unfortunately, comparisons for significance in survival time in this study cannot be made since there was no active control group (Balint et al., 2015).

### Ad5-PSA

Prostatic epithelial cells secrets prostate specific antigens (PSA), which belongs to tissue kallikrein-related peptidase family (Prassas et al., 2015). PSA is considered a target for prostate cancer treatment because it is exclusively derived from prostate tissue and participates in prostate cancer signaling pathways such as angiogenesis, invasion, and tumor microenvironment regulation (Moradi et al., 2019).

In a murine prostate cancer model, Ad5 with the insertion of a full-length PSA gene (Ad5-PSA) activated a strong anti-PSA immune response that suppressed growth rate of PSA-generating tumor (Elzey et al., 2001). Lubaroff et al. (2009) treated 32 patients with hormone-refractory metastatic prostate cancer by injecting a single dose of an Ad5-PSA vaccine. This vaccine was proven safe with no serious treatment-related AEs. Thirty-four percent of vaccinated patients produced anti-PSA antibodies and 68% produced anti-PSA T-cell responses. Notably, 55% of patients survived longer than expected survival times calculated using Halabi nomogram (Lubaroff et al., 2009). In order to determine the therapeutic efficacy of the vaccine, researchers enrolled 81 patients with recurrent or hormone-refractory prostate cancer in a phase II clinical trial (NCT00583024) (Lubaroff et al., 2012). The preliminary results showed that all of the patients with recurrent prostate cancer and 67% of patients with hormone-refractory prostate cancer had anti-PSA immune responses above the levels detected prior to vaccination (Lubaroff et al., 2012). However, this trial that determine the PFS and OS in these patients would necessitate many years of observation because of the slow growth of prostate cancer cells.

### Coadministration of Multiple Types of Cancer Vaccines

The potential advantages of adenoviruses as basis for cancer vaccines have been proven in several preclinical and clinical trials (Majhen et al., 2014). Although these findings are certainly encouraging, the majority of studies remains in phase I/II trials where adenovirus-based vaccines still show few treatment outcomes in advanced cancer patients. Tumor heterogeneity and low immunogenicity of target antigens may be the main reason for constraining the development of adenovirus-based cancer vaccines. To overcome these barriers, coadministration of multiple types of cancer vaccines has been proposed.

Elzey et al. (2001) demonstrated that large (500–1,000 mm^3^) established prostate tumors in mice were efficiently eliminated by injection of Ad5-PSA in combination with recombinant canarypox viruses (ALVAC) encoding IL-12, IL-2, or TNF-α 7 days later. Melanoma-associated antigen 3 (MAGE-A3) is specifically expressed in the placenta and germline cells of the testis but frequently overexpressed in sarcoma and other tumor tissues (Conley et al., 2019). Maraba virus belongs to the vesiculovirus genus of the *Rhabdoviridae* family and MG1 strain of the Maraba virus has shown oncolytic activity in numerous preclinical cancer models (Pol et al., 2018). Pol et al. (2019) constructed adenovirus and Maraba virus vectors to express human MAGE-A3 (Ad-MAGEA3, MG1-MAGEA3), leading to engage multiple effector immune cells against hMAGEA3 in macaques. Boosting with MG1-MAGEA3, Ad-MAGEA3 induced the expansion of hMAGE-A3-specific CD4^+^ and CD8^+^ T cells, which reached a peak and persisted for several months (Pol et al., 2019). Using the same strategies, two phase I/II clinical trials are underway to evaluate the ability of the Ad:MG1 approach for clinical treatment. The first trial utilized MG1-MAGEA3 with or without Ad-MAGEA3 to treat patients with advanced MAGE-A3-expressing solid tumors (NCT02285816). The second one tested MG1-MAGEA3 with Ad-MAGEA3 in patients with non-small cell lung cancer (NSCLC) combined with pembrolizumab, an anti-PD-L1 antibody (NCT02879760). An open-label phase I trial conducted by Gatti-Mays et al. (2020) (NCT03384316) enrolled nine patients with colorectal cancer and one with cholangiocarcinoma to evaluate the synergic effect of three therapeutic vaccines (ETBX-011, ETBX-061, and ETBX-051). These vaccines based on the Ad5 [E1-, E2b-] platform targeted three TAAs—CEA, MUC-1, and brachyury, respectively. Antigen-specific T cells to at least one TAA encoded by vaccines were generated in all patients, and 67% of them developed CEA-specific T-cell responses after vaccination. There were only temporary treatment-related AEs, including injection site reactions and flu-like symptoms (Gatti-Mays et al., 2020). Recently, Bilusic et al. (2021) combined three therapeutic vaccines (ETBX-071, ETBX-061, ETBX-051) to treat 18 patients with metastatic castration-resistant prostate cancer (NCT03481816). ETBX-071 is a PSA-targeting vaccine that employs Ad5 [E1-, E2b-] platform inserting a PSA gene (Ad5 [E1-, E2b-]-PSA). There was no grade > 3 treatment-related AEs or DLTs in all patients, indicating that these three vaccines were tolerable and safe. The median PFS was 22 weeks and 47% of patients mounted immune responses to PSA, MUC-1, and brachyury (Bilusic et al., 2021).

## Oncolytic Adenovirus and Immune Checkpoint Inhibitors

Immune checkpoints refer to the set of inhibitory pathways that limit collateral tissues damage when the immune system is responding to pathogenic infection and also maintain self-tolerance to prevent autoimmune diseases (Pardoll, 2012; Abril-Rodriguez and Ribas, 2017). Tumor cells disguise themselves as regular components of the human body to escape from immune elimination by immune checkpoint pathways (Li et al., 2019). Therefore, the blockades of the immune checkpoints can unleash the brake of the immune system and induce antitumor immune responses (Abril-Rodriguez and Ribas, 2017). Immune checkpoint inhibitors (ICIs), which block immune checkpoints to enhance T-cell-mediated immune responses against cancer cells, have substantially improved clinical outcomes in multiple malignancies (Sharma and Allison, 2015). Major targeted immune checkpoints include cytotoxic T lymphocyte-associated antigen 4 (CTLA-4), PD-1, and its ligand, PD-L1. Activation of T cells results in accumulation of CTLA-4 at the interface between T cell and APC, reaching a level where it opposes CD28 costimulation and abrogates an activated T-cell response (Sharma and Allison, 2015). On the other hand, PD-1 and PD-L1 protect malignant cells from T-cell attack *via* interfering with signaling mediated by the T cell antigen receptor (Han et al., 2020). Given that anti-CTLA-4 therapy and anti-PD-1/PD-L1 therapy only benefits a fraction of patients because of the immunosuppressive tumor microenvironment, there are ongoing efforts to reverse immune suppression and create a more favorable microenvironment for ICIs. To achieve this, combining OAds and ICIs is a potent approach. Table 3 lists clinical trials of OAds in combination with ICIs.

**TABLE 3:** Clinical trials on OAds in combination with immune checkpoint inhibitors.

OAd name	Transgene	Combination therapy	Indication	Trial number	Phase
ONCOS-102 (Ad5/3-D24-GM-CSF)	GM-CSF	Pembrolizumab, cyclophosphamide	Melanoma	NCT03003676	I
		Durvalumab	Colorectal cancer, ovarian cancer, appendiceal cancer	NCT02963831	I/II
Delta-24-RGD (DNX-2401)		Pembrolizumab	Glioblastoma, gliosarcoma	NCT02798406	II
LOAd703	CD40L, 4-1BBL	Gemcitabine, Nab-paclitaxel, atezolizumab	Pancreatic cancer	NCT02705196	I/II
		Atezolizumab	Melanoma	NCT04123470	I/II
VCN-01 (Ad-E2F-D24RGD-PH20)	PH-20	Durvalumab	Head and neck squamous cell carcinoma	NCT03799744	I
OBP-301		Pembrolizumab	Advanced solid tumor	NCT03172819	I
		Pembrolizumab	Esophagogastric adenocarcinoma	NCT03921021	II
CG0070	GM-CSF	Pembrolizumab	Non-muscle invasive bladder cancer	NCT04387461	II
Ad-MAGEA3	MAGE-A3	MG1-MAGEA3, pembrolizumab	Non-small-cell lung cancer	NCT02879760	I/II

### ONCOS-102

As noted above, ONCOS-102 is an OAd-encoding GM-CSF transgene, and it was found to correlate with upregulated PD-L1 expression in mesothelioma patients by Ranki et al. (2016). Pembrolizumab is a humanized antibody suppressing the PD-1/PD-L1-mediated interference of T-cell signaling. Kuryk et al. (2019) observed synergistic antitumor effects in melanoma-engrafted mice treated with the combination of ONCOS-102 with pembrolizumab. It is noteworthy that this combination strategy could be more effective in reducing tumor volume than pembrolizumab alone, suggesting that ONCOS-102 may promote the therapeutic effects of pembrolizumab synergistically. These findings provided the scientific rationale for a study to investigate the efficacy of combination therapy for melanoma patients who showed progression after PD-1 blockade (NCT03003676) (Kuryk et al., 2019). Shoushtari et al. reported the efficacy and safety of ONCOS-102 in combination with pembrolizumab in nine patients with advanced melanoma (NCT02879669) on the 34th Annual Meeting and Preconference Programs of the Society for Immunotherapy of Cancer. A remarkable rise in proinflammtory cytokines and circulating CD8^+^ T cells was observed in all patients. Thirty-three percent of the patients achieved complete or partial responses according to the Response Evaluation Criteria in Solid Tumors (RECIST) 1.1. There were no DLTs, and the most common AEs were chills and fever associated with Ad replication. Disappointingly, one patient was diagnosed with infectious colitis related to ONCOS-102.

Furthermore, an ongoing phase I/II study combines ONCOS-102 with durvalumab, an anti-PD-L1 antibody, for the treatment of advanced peritoneal malignancies (NCT02963831). The combination therapy seems to be a promising strategy to activate immune responses in anti-PD-1/PD-L1 refractory cancers.

### Delta-24-RGD and Delta-24-RGDOX

Delta-24-RGD is an engineered adenovirus with a 24-base pair deletion and an insertion of the RGD-4C peptide motif into the adenoviral fiber knob. These modifications allowed Delta-24-RGD to replicate more efficiently in low-CXADR-expressing glioma cell lines (Fueyo et al., 2003). Delta-24-RGD treatment elicited antitumor effects and correlated with longer survival in preclinical models of pancreatic ductal adenocarcinoma (PDAC) (Dai et al., 2017), gliomas (Martínez-Vélez et al., 2019), atypical teratoid/rhabdoid tumor (AT/RT), and central nervous system primitive neuroectodermal tumor (CNS-PNET) (Garcia-Moure et al., 2020). A phase I clinical trial of Delta-24-RGD was conducted in 37 patients with recurrent malignant glioma (NCT00805376) (Lang et al., 2018). Of the 25 patients receiving single Delta-24-RGD administration, 20% survived more than 3 years, and three patients had over 95% reduction in tumor volumes. No DLTs were observed in this study and only two patients experienced grades 1 to 2 AEs related to Delta-24-RGD (Lang et al., 2018). Analyses of posttreatment surgical specimens showed that transmembrane immunoglobulin mucin-3 (TIM-3) was downregulated, which indicated that Delta-24-RGD may partially overcome T-cell exhaustion and thus relieve tumor immunosuppression (Wherry and Kurachi, 2015; Lang et al., 2018). Delta-24-RGD is now tested in a phase II clinical trial with pembrolizumab in 48 patients with recurrent glioblastoma or gliosarcoma (NCT02798406) (Aiken et al., 2019). The medium OS of patients treated with delta-24-RGD and pembrolizumab was 12 months, and three patients were alive more than 20 months. Forty-seven percent of patients experienced stable disease or better, and two had over 94% regression of tumor from Delta-24-RGD administration. The majority of AEs were mild to moderate and unrelated to Delta-24-RGD (Aiken et al., 2019). However, Aiken et al. (2019) did not assign patients to Delta-24-RGD or pembrolizumab monotherapy groups. Owing to the lack of comparison between combination therapy and monotherapy, the effect of Delta-24-RGD to the pembrolizumab monotherapy is difficult to interpret.

Delta-24-RGDOX is a variant based on the virus backbone of Delta-24-RGD, which expresses immune costimulator OX40L to induce superior tumor-specific immunity. Since Delta-24-RGDOX injection induced upregulation of PD-L1 expression on the glioma cells, Jiang et al. (2017) combined this virus with an anti-PD-L1 antibody to overcome immunosuppression mediated by PD-L1 expression, resulting in a long-term survival rate of 85%. In the brains of the long-term surviving mice, complete tumor regression was observed at the tumor implantation site. Thus, Delta-24-RGDOX in combination with an anti-PD-L1 antibody seems to have induced the formation of immunological memory that prevented tumor growth (Jiang et al., 2017).

### Ad5/3-D24aCTLA4

Anti-CTLA4 antibodies can prevent the dysfunction of T cells and potentiate tumor-specific immune responses by blocking the activity of CTLA-4 (Sharma and Allison, 2015). Promising efficacy of anti-CTLA4 antibodies in cancer immunotherapy have already been demonstrated in several clinical trials, whereas severe immune-related AEs occurred when normal cells were exposed to these agents (Hodi et al., 2010). Currently, local administration of anti-CTLA4 antibodies is a feasible strategy to increase concentration at the target while reducing systemic AEs (Aiken et al., 2019).

Subsequently, an OAd expressing a complete human anti-CTLA4 monoclonal antibody (Ad5/3-D24aCTLA4) was designed (Dias et al., 2012). T-cell activation and direct proapoptotic effects mediated by anti-CTLA4 antibodies were observed in PBMC of patients with advanced solid tumors but not those of normal donors. Local expression of anti-CTLA4 antibodies resulted in 43-fold higher concentrations in tumor than plasma (Dias et al., 2012).

### OAds Expressing Anti-PD-L1 Antibody

Anti-PD-L1 antibodies have been extensively investigated to improve durable response rate and overall survival of advanced cancer (Xia et al., 2019). Tanoue et al. (2017) developed a combinatorial adenovirus vector system which consists of an oncolytic adenovirus and a helper-dependent adenovirus expressing anti-PD-L1 antibodies (CAd-VECPDL1). In prostate cancer xenograft mouse models, local production of PD-L1 blocking mini-antibodies by CAd-VECPDL1 in combination with tumor-directed CAR T cells induced more potent antitumor effects against bulky solid tumors than systemic infusion of anti-PD-L1 IgG and CAR T cells (Tanoue et al., 2017). Chimeric antigen receptor (CAR) T-cell therapy entails the genetic modification of patient-derived T cells to express a CAR that is designed to recognize tumor antigens and elicit tumor-specific T-cell activation (Kosti et al., 2018). Compared with physiologic T-cell receptor (TCRs), the CARs can recognize more extensive tumor antigens independently of their expression of major histocompatibility antigens (Ma et al., 2019). The activation of T cells is accomplished by the intracellular signaling domain of CARs containing CD3ζ chain linked with zero or one or two costimulatory molecules such as CD28, CD137, and CD134 (Wang Z. et al., 2017). Although CAR T-cell therapy has produced striking clinical successes in the treatment of hematologic malignancies, it has shown limited responses in solid tumors (Kosti et al., 2018). Tanoue et al. (2017) demonstrated that the combination of CAd-VECPDL1 and CAR T cells augmented the functionality of CAR T cells against solid tumors and yielded oncolysis at the tumor site. For optimizing effector function, Rosewell Shaw et al. (2017) further modified CAd-VECPDL1 by adding an IL-12p70 transgene (CAd12_PDL1) and found that combining local administration of CAd12_PDL1 with systemic CAR T-cell infusion prolonged survival in head and neck squamous cell carcinoma (HNSCC) xenograft models and controlled the growth of both primary and metastasized tumors in orthotopic models of HNSCC. Bispecific tumor-targeted T-cell engager (BiTE) molecules comprise two single-chain variable fragment (scFv) regions, one of which targets tumor-expressed antigens and another is specific for CD3, the invariable part of TCR, leading to redirection of T cells to tumor cells (Einsele et al., 2020). However, one characteristic of BiTE molecules is their short serum half-life (Einsele et al., 2020). CD44 variant 6 (CD44v6) is a marker of cancer stem cells driving metastasis and is highly expressed in tumor cells, with little expression in normal tissues (Todaro et al., 2014). Porter et al. (2020) incorporated a BiTE molecule targeting CD44v6 into a CAd-encoding IL-12p70 and anti-PD-L1 antibody (CAdTrio) to warrant high BiTE levels in tumors of murine xenograft models. CAdtrio enhanced the early and long-term antitumor activity of CAR T cells and suppress tumor growth in xenograft mouse models through expressing CD44v6.BiTE molecules, anti-PD-L1 antibodies, and IL-12 (Porter et al., 2020). In addition, clinical trials are ongoing to investigate the combinatorial strategies involving OAds and ICIs. LOAd703 is intratumorally administered to patients with PDAC (NCT02705196) and malignant melanoma (NCT04123470) combined with atezolizumab, an anti-PD-L1 antibody. VCN-01 (Ad-E2F-D24RGD-PH20) is a genetically modified OAd characterized by the selective replication in tumor cells with high abundance of free E2F-1 and expression of human hyaluronidase (PH-20) that enhanced intratumor spread of the virus (Pascual-Pasto et al., 2019). Combination of VCN-01 with durvalumab is evaluated in patients with recurrent or metastatic HNSCC (NCT03799744). Moreover, a telomerase-specific OAd (OBP-301) with hTERT promoter which can regulate the expression of E1A and E1B genes has shown direct and distant antitumor effects in a mouse model of prostate cancer and renal cell carcinoma (Huang et al., 2008, 2010). OBP-301 is currently utilized in a phase I study for patients with advanced solid tumors combining with pembrolizumab (NCT03172819). A phase II study of same combination therapy is tested in patients with advanced gastric and gastroesophageal junction adenocarcinoma who had progressed prior therapy (NCT03921021). Furthermore, Kanaya et al. (2020) constructed OBP-502 by adding the gene cassette expressing RGD peptide in the E3 region of OBP-301. OBP-502 in combination with an anti-PD-1 antibody significantly suppressed the tumor growth in CT26 and PAN02 bilateral subcutaneous tumor models (Kanaya et al., 2020).

## Challenges and Solutions

In spite of superior antitumor effectiveness and tolerable safety profiles, current oncolytic adenovirus immunotherapy still faces challenges that limit the clinical application of it. To overcome the limitations that include antiviral immune response and obstruction of TME, some potential solutions are provided.

Most individuals have preexisting neutralizing antibodies (NAbs) against different adenovirus types, because these viruses are the cause of common respiratory infections (Yu et al., 2012). Antiviral immune response mediated by NAbs remains a major challenge in cancer immunotherapy with recombinant Ad-based vectors, especially wild-type Ad5, as it can mitigate their therapeutic capability, even leading to early therapy termination (Balint et al., 2015). Substantial exposure to virus such as systemic administration and multiple-dose regimens results in high NAbs titers (Hemminki et al., 2012), and thus most OAds are injected intratumorally to overwhelm antiviral mechanisms. Alternatively, other strategies have been utilized to avoid antibody neutralization including switching Ad type (Hemminki et al., 2012), alternations in the Ad5 viral capsid (Roy et al., 1998), reductions in viral protein expression (Morse et al., 2013), and polymer-coated adenovirus (Wang C.-H. K. et al., 2011). A recent study has reported a bifunctional adapter harboring the DE1 domain of the adenovirus hexon and an established polysialic acid-specific scFv as a tumor-specific ligand (Niemann et al., 2019). This bifunctional adapter can retarget anti-adenoviral NAbs to tumor cells for amplifying the therapeutic effectiveness and providing a novel strategy to further exploit the potential of oncolytic virotherapy (Niemann et al., 2019). Of note, the question of Nabs is complex. The presence of neutralizing antiviral antibodies before treatment seems to correlate with shorter survival of cancer patients (Taipale et al., 2016). In contrast, NAbs had no effect on survival duration in another study (Heo et al., 2013). This is probably because the baseline antibodies titers are often at low levels, thus NAbs may be unable to completely block OAds when large amounts of virus are given in a typical treatment.

Additionally, the TME represents as an obstruction against virotherapy. In solid tumors, TME is composed of a complex mixture of malignant cells and non-malignant tissues that contain myeloid-derived suppressor cells, fibroblasts, endothelial cells, and extracellular matrix (ECM) (Tian et al., 2019). The dissemination of OAds throughout the entire tumor mass is impeded by accumulation of fibroblasts, dense ECM, and formation of neovasculature (Yamamoto et al., 2017). Therefore, OAds targeting the ECM (Vera et al., 2016) and angiogenesis (Choi et al., 2015) have been generated to overcome this physical barrier. Based on Ad3 fiber protein, Yumul et al. (2016) developed a self-dimerizing recombinant protein to trigger the transient opening of epithelial junctions, thus breaching barriers of epithelial cells and facilitating lateral virus spread. Furthermore, owing to irregular structure of blood vessels in tumors, the overall blood perfusion rates in tumors are reduced, leaving poorly perfused or even unperfused regions (Jain and Stylianopoulos, 2010). The inadequate perfusion causes a hostile TME including hypoxia, low pH, and necrotic tissue, which leads to drug resistance and tumor progression (Jain and Stylianopoulos, 2010). Some of these obstacles have been addressed while others still lack proper solutions. Lu et al. (2015) constructed a potent OAd driven by hypoxia response element, retaining its anti-tumor activity even in significant areas of hypoxia. Recently, a hypoxia-responsive and cancer-specific modified human telomerase reverse transcriptase (H5CmTERT) promoter was generated to regulate replication of an OAd (H5CmTERT-Ad) even under the hypoxia environment (Oh et al., 2018).

## Conclusion

With increasing numbers of genomic modification strategies, impressive preclinical and clinical outcomes for modified oncolytic adenoviruses are continuously emerging, and multiple clinical trials are ongoing. OAds induce direct oncolysis and upregulate the immunostimulatory signals intratumorally, which can dramatically reduce local immunosuppression and enhance tumor-specific immunity. Good safety and tolerability of OAds have been confirmed in most extant clinical trials. Nevertheless, virtually no monotherapy will likely defeat all immune evasion mechanisms. OAds in combination with immune checkpoint inhibitors provides a promising approach to achieve long-term tumor control in patients who are unresponsive to systemic immune checkpoint blockade. Further research of OAd requires more attention for confronting the challenges, thus breaking its limitations and maximizing efficacy of treatment with minimal systemic toxicity.

## Author Contributions

YZ, ZL, LL, JW, and HuZ: original draft preparation. ZL, LL, TL, and HaZ: manuscript reviewing and editing. BX: project administration and final manuscript supervision. All authors contributed to the article and approved the submitted version.

## Conflict of Interest

The authors declare that the research was conducted in the absence of any commercial or financial relationships that could be construed as a potential conflict of interest.

## References

[B1] Abril-Rodriguez G. Ribas A. (2017). SnapShot: Immune Checkpoint Inhibitors. *Cancer Cell* 31:010. 10.1016/j.ccell.2017.05.010 28609660

[B2] Aiken R. Chen C. Cloughesy T. Colman H. Daras M. Groves M. (2019). Interim results of a phase II multi-center study of oncolytic adenovirus DNX-2401 with pembrolizumab for recurrent glioblastoma; captive study (KEYNOTE-192). *Neuro Oncol.* 21 8–9.

[B3] Alderson M. R. Smith C. A. Tough T. W. Davis-Smith T. Armitage R. J. Falk B. (1994). Molecular and biological characterization of human 4-1BB and its ligand. *Eur. J. Immunol.* 24 2219–2227. 8088337 10.1002/eji.1830240943

[B4] Andtbacka R. H. I. Kaufman H. L. Collichio F. Amatruda T. Senzer N. Chesney J. (2015). Talimogene Laherparepvec Improves Durable Response Rate in Patients With Advanced Melanoma. *J. Clin. Oncol.* 33 2780–2788. 10.1200/JCO.2014.58.3377 26014293

[B5] Arnberg N. (2012). Adenovirus receptors: implications for targeting of viral vectors. *Trends Pharmacol. Sci.* 33 442–448. 10.1016/j.tips.2012.04.005 22621975

[B6] Baker A. T. Greenshields-Watson A. Coughlan L. Davies J. A. Uusi-Kerttula H. Cole D. K. (2019). Diversity within the adenovirus fiber knob hypervariable loops influences primary receptor interactions. *Nat. Commun.* 10:741. 10.1038/s41467-019-08599-y 30765704 PMC6376029

[B7] Balint J. P. Gabitzsch E. S. Rice A. Latchman Y. Xu Y. Messerschmidt G. L. (2015). Extended evaluation of a phase 1/2 trial on dosing, safety, immunogenicity, and overall survival after immunizations with an advanced-generation Ad5 [E1-, E2b-]-CEA(6D) vaccine in late-stage colorectal cancer. *Cancer Immunol. Immunother.* 64 977–987. 10.1007/s00262-015-1706-4 25956394 PMC4506904

[B8] Barrett J. A. Cai H. Miao J. Khare P. D. Gonzalez P. Dalsing-Hernandez J. (2018). Regulated intratumoral expression of IL-12 using a RheoSwitch Therapeutic System (RTS) gene switch as gene therapy for the treatment of glioma. *Cancer Gene Ther.* 25 106–116. 10.1038/s41417-018-0019-0 29755109 PMC6021367

[B9] Bauerschmitz G. J. Guse K. Kanerva A. Menzel A. Herrmann I. Desmond R. A. (2006). Triple-targeted oncolytic adenoviruses featuring the cox2 promoter, E1A transcomplementation, and serotype chimerism for enhanced selectivity for ovarian cancer cells. *Mol. Ther.* 14 164–174. 10.1016/j.ymthe.2006.01.010 16580264

[B10] Bilusic M. McMahon S. Madan R. A. Karzai F. Tsai Y.-T. Donahue R. N. (2021). Phase I study of a multitargeted recombinant Ad5 PSA/MUC-1/brachyury-based immunotherapy vaccine in patients with metastatic castration-resistant prostate cancer (mCRPC). *J. Immunother. Cancer* 9:002374. 10.1136/jitc-2021-002374 33762322 PMC7993215

[B11] Bischoff J. R. Kirn D. H. Williams A. Heise C. Horn S. Muna M. (1996). An adenovirus mutant that replicates selectively in p53-deficient human tumor cells. *Science* 274 373–376. 10.1126/science.274.5286.373 8832876

[B12] Bramante S. Kaufmann J. K. Veckman V. Liikanen I. Nettelbeck D. M. Hemminki O. (2015). Treatment of melanoma with a serotype 5/3 chimeric oncolytic adenovirus coding for GM-CSF: Results in vitro, in rodents and in humans. *Int. J. Cancer* 137 1775–1783. 10.1002/ijc.29536 25821063

[B13] Burke J. M. Lamm D. L. Meng M. V. Nemunaitis J. J. Stephenson J. J. Arseneau J. C. (2012). A first in human phase 1 study of CG0070, a GM-CSF expressing oncolytic adenovirus, for the treatment of nonmuscle invasive bladder cancer. *J. Urol.* 188 2391–2397. 10.1016/j.juro.2012.07.097 23088985

[B14] Cao G.-D. He X.-B. Sun Q. Chen S. Wan K. Xu X. (2020). The Oncolytic Virus in Cancer Diagnosis and Treatment. *Front. Oncol.* 10:1786. 10.3389/fonc.2020.01786 33014876 PMC7509414

[B15] Cervera-Carrascon V. Siurala M. Santos J. M. Havunen R. Tähtinen S. Karell P. (2018). TNFa and IL-2 armed adenoviruses enable complete responses by anti-PD-1 checkpoint blockade. *Oncoimmunology* 7:e1412902. 10.1080/2162402X.2017.1412902 29721366 PMC5927535

[B16] Chaurasiya S. Yang A. Kang S. Lu J. Kim S.-I. Park A. K. (2020). Oncolytic poxvirus CF33-hNIS-ΔF14.5 favorably modulates tumor immune microenvironment and works synergistically with anti-PD-L1 antibody in a triple-negative breast cancer model. *Oncoimmunology* 9:1729300. 10.1080/2162402X.2020.1729300 32158622 PMC7051185

[B17] Chen R.-F. Lee C.-Y. (2014). Adenoviruses types, cell receptors and local innate cytokines in adenovirus infection. *Int. Rev. Immunol.* 33 45–53. 10.3109/08830185.2013.823420 24127823

[B18] Chen W. Wu Y. Liu W. Wang G. Wang X. Yang Y. (2011). Enhanced antitumor efficacy of a novel fiber chimeric oncolytic adenovirus expressing p53 on hepatocellular carcinoma. *Cancer Lett.* 307:021. 10.1016/j.canlet.2011.03.021 21504839

[B19] Chester C. Sanmamed M. F. Wang J. Melero I. (2018). Immunotherapy targeting 4-1BB: mechanistic rationale, clinical results, and future strategies. *Blood* 131 49–57. 10.1182/blood-2017-06-741041 29118009

[B20] Chin S. M. Kimberlin C. R. Roe-Zurz Z. Zhang P. Xu A. Liao-Chan S. (2018). Structure of the 4-1BB/4-1BBL complex and distinct binding and functional properties of utomilumab and urelumab. *Nat. Commun.* 9:4679. 10.1038/s41467-018-07136-7 30410017 PMC6224509

[B21] Chiocca E. A. Yu J. S. Lukas R. V. Solomon I. H. Ligon K. L. Nakashima H. (2019). Regulatable interleukin-12 gene therapy in patients with recurrent high-grade glioma: Results of a phase 1 trial. *Sci. Transl. Med.* 11:aaw5680. 10.1126/scitranslmed.aaw5680 31413142 PMC7286430

[B22] Choi I.-K. Shin H. Oh E. Yoo J. Y. Hwang J. K. Shin K. (2015). Potent and long-term antiangiogenic efficacy mediated by FP3-expressing oncolytic adenovirus. *Int. J. Cancer* 137 2253–2269. 10.1002/ijc.29592 25944623

[B23] Colombo M. P. Trinchieri G. (2002). Interleukin-12 in anti-tumor immunity and immunotherapy. *Cytokine Growth Factor Rev.* 13 155–168. 10.1016/s1359-6101(01)00032-6 11900991

[B24] Conley A. P. Wang W.-L. Livingston J. A. Ravi V. Tsai J.-W. Ali A. (2019). MAGE-A3 is a Clinically Relevant Target in Undifferentiated Pleomorphic Sarcoma/Myxofibrosarcoma. *Cancers* 11:cancers11050677. 10.3390/cancers11050677 31096717 PMC6562561

[B25] Curran C. S. Evans M. D. Bertics P. J. (2011). GM-CSF production by glioblastoma cells has a functional role in eosinophil survival, activation, and growth factor production for enhanced tumor cell proliferation. *J. Immunol.* 187 1254–1263. 10.4049/jimmunol.1001965 21705618 PMC3140612

[B26] Dai B. Roife D. Kang Y. A. Gumin J. Rios Perez M. V. Li X. (2017). Preclinical Evaluation of Sequential Combination of Oncolytic Adenovirus Delta-24-RGD and Phosphatidylserine-Targeting Antibody in Pancreatic Ductal Adenocarcinoma. *Mol. Cancer Ther.* 16 662–670. 10.1158/1535-7163.MCT-16-0526 28138026 PMC5512885

[B27] Dias J. D. Hemminki O. Diaconu I. Hirvinen M. Bonetti A. Guse K. (2012). Targeted cancer immunotherapy with oncolytic adenovirus coding for a fully human monoclonal antibody specific for CTLA-4. *Gene Ther.* 19 988–998. 10.1038/gt.2011.176 22071969

[B28] Do M.-H. To P. K. Cho Y.-S. Kwon S.-Y. Hwang E. C. Choi C. (2018). Targeting CD46 Enhances Anti-Tumoral Activity of Adenovirus Type 5 for Bladder Cancer. *Int. J. Mol. Sci.* 19:ijms19092694. 10.3390/ijms19092694 30201920 PMC6164063

[B29] Einsele H. Borghaei H. Orlowski R. Z. Subklewe M. Roboz G. J. Zugmaier G. (2020). The BiTE (bispecific T-cell engager) platform: Development and future potential of a targeted immuno-oncology therapy across tumor types. *Cancer* 126 3192–3201. 10.1002/cncr.32909 32401342

[B30] Elzey B. D. Siemens D. R. Ratliff T. L. Lubaroff D. M. (2001). Immunization with type 5 adenovirus recombinant for a tumor antigen in combination with recombinant canarypox virus (ALVAC) cytokine gene delivery induces destruction of established prostate tumors. *Int. J. Cancer* 94 842–849. 10.1002/ijc.1556 11745487

[B31] Eriksson E. Milenova I. Wenthe J. Ståhle M. Leja-Jarblad J. Ullenhag G. (2017a). Shaping the Tumor Stroma and Sparking Immune Activation by CD40 and 4-1BB Signaling Induced by an Armed Oncolytic Virus. *Clin. Cancer Res.* 23 5846–5857. 10.1158/1078-0432.CCR-17-0285 28536305

[B32] Eriksson E. Moreno R. Milenova I. Liljenfeldt L. Dieterich L. C. Christiansson L. (2017b). Activation of myeloid and endothelial cells by CD40L gene therapy supports T-cell expansion and migration into the tumor microenvironment. *Gene Ther.* 24:80. 10.1038/gt.2016.80 27906162 PMC5441514

[B33] Evgin L. Huff A. L. Wongthida P. Thompson J. Kottke T. Tonne J. (2020). Oncolytic virus-derived type I interferon restricts CAR T cell therapy. *Nat. Commun.* 11:3187. 10.1038/s41467-020-17011-z 32581235 PMC7314766

[B34] Fueyo J. Alemany R. Gomez-Manzano C. Fuller G. N. Khan A. Conrad C. A. (2003). Preclinical characterization of the antiglioma activity of a tropism-enhanced adenovirus targeted to the retinoblastoma pathway. *J. Natl. Cancer Inst.* 95 652–660. 10.1093/jnci/95.9.652 12734316

[B35] Fusciello M. Fontana F. Tähtinen S. Capasso C. Feola S. Martins B. (2019). Artificially cloaked viral nanovaccine for cancer immunotherapy. *Nat. Commun.* 10:5747. 10.1038/s41467-019-13744-8 31848338 PMC6917704

[B36] Gabitzsch E. S. Tsang K. Y. Palena C. David J. M. Fantini M. Kwilas A. (2015). The generation and analyses of a novel combination of recombinant adenovirus vaccines targeting three tumor antigens as an immunotherapeutic. *Oncotarget* 6 31344–31359. 10.18632/oncotarget.5181 26374823 PMC4741610

[B37] Gao J. Zhang W. Ehrhardt A. (2020a). Expanding the Spectrum of Adenoviral Vectors for Cancer Therapy. *Cancers* 12:cancers12051139. 10.3390/cancers12051139 32370135 PMC7281331

[B38] Gao J. Zhang W. Mese K. Bunz O. Lu F. Ehrhardt A. (2020b). Transient Chimeric Ad5/37 Fiber Enhances NK-92 Carrier Cell-Mediated Delivery of Oncolytic Adenovirus Type 5 to Tumor Cells. *Mol. Ther. Methods Clin. Dev.* 18 376–389. 10.1016/j.omtm.2020.06.010 32695840 PMC7358217

[B39] Garcia-Moure M. Gonzalez-Huarriz M. Labiano S. Guruceaga E. Bandres E. Zalacain M. (2020). Delta-24-RGD, an Oncolytic Adenovirus, Increases Survival and Promotes Proinflammatory Immune Landscape Remodeling in Models of AT/RT and CNS-PNET. *Clin. Cancer Res.* 2020:3313. 10.1158/1078-0432.CCR-20-3313 33376098 PMC7617079

[B40] Gatti-Mays M. E. Redman J. M. Collins J. M. Bilusic M. (2017). Cancer vaccines: Enhanced immunogenic modulation through therapeutic combinations. *Hum. Vaccin. Immunother.* 13 2561–2574. 10.1080/21645515.2017.1364322 28857666 PMC5703410

[B41] Gatti-Mays M. E. Redman J. M. Donahue R. N. Palena C. Madan R. A. Karzai F. (2020). A Phase I Trial Using a Multitargeted Recombinant Adenovirus 5 (CEA/MUC1/Brachyury)-Based Immunotherapy Vaccine Regimen in Patients with Advanced Cancer. *Oncologist* 25:0608. 10.1634/theoncologist.2019-0608 31594913 PMC7288633

[B42] Goodwin R. G. Din W. S. Davis-Smith T. Anderson D. M. Gimpel S. D. Sato T. A. (1993). Molecular cloning of a ligand for the inducible T cell gene 4-1BB: a member of an emerging family of cytokines with homology to tumor necrosis factor. *Eur. J. Immunol.* 23 2631–2641. 10.1002/eji.1830231037 8405064

[B43] Gujar S. Pol J. G. Kroemer G. (2018b). Heating it up: Oncolytic viruses make tumors ‘hot’ and suitable for checkpoint blockade immunotherapies. *Oncoimmunology* 7:e1442169. 10.1080/2162402X.2018.1442169 30221036 PMC6136862

[B44] Gujar S. Pol J. G. Kim Y. Lee P. W. Kroemer G. (2018a). Antitumor Benefits of Antiviral Immunity: An Underappreciated Aspect of Oncolytic Virotherapies. *Trends Immunol.* 39 209–221. 10.1016/j.it.2017.11.006 29275092

[B45] Hall K. Blair Zajdel M. E. Blair G. E. (2009). Defining the role of CD46, CD80 and CD86 in mediating adenovirus type 3 fiber interactions with host cells. *Virology* 392 222–229. 10.1016/j.virol.2009.07.010 19682720

[B46] Han Y. Liu D. Li L. (2020). PD-1/PD-L1 pathway: current researches in cancer. *Am. J. Cancer Res.* 10 727–742. 32266087 PMC7136921

[B47] Havunen R. Siurala M. Sorsa S. Grönberg-Vähä-Koskela S. Behr M. Tähtinen S. (2017). Oncolytic Adenoviruses Armed with Tumor Necrosis Factor Alpha and Interleukin-2 Enable Successful Adoptive Cell Therapy. *Mol. Ther. Oncolyt.* 4 77–86. 10.1016/j.omto.2016.12.004 28345026 PMC5363700

[B48] Hegde P. S. Chen D. S. (2020). Top 10 Challenges in Cancer Immunotherapy. *Immunity* 52 17–35. 10.1016/j.immuni.2019.12.011 31940268

[B49] Heise C. Hermiston T. Johnson L. Brooks G. Sampson-Johannes A. Williams A. (2000). An adenovirus E1A mutant that demonstrates potent and selective systemic anti-tumoral efficacy. *Nat. Med.* 6 1134–1139. 10.1038/80474 11017145

[B50] Hemminki O. Hemminki A. (2016). A century of oncolysis evolves into oncolytic immunotherapy. *Oncoimmunology* 5:e1074377. 10.1080/2162402x.2015.1074377 27057442 PMC4801451

[B51] Hemminki O. Diaconu I. Cerullo V. Pesonen S. K. Kanerva A. Joensuu T. (2012). Ad3-hTERT-E1A, a fully serotype 3 oncolytic adenovirus, in patients with chemotherapy refractory cancer. *Mol. Ther.* 20 1821–1830. 10.1038/mt.2012.115 22871667 PMC3437574

[B52] Hemminki O. Parviainen S. Juhila J. Turkki R. Linder N. Lundin J. (2015). Immunological data from cancer patients treated with Ad5/3-E2F-Δ24-GMCSF suggests utility for tumor immunotherapy. *Oncotarget* 6 4467–4481. 10.18632/oncotarget.2901 25714011 PMC4414204

[B53] Heo J. Reid T. Ruo L. Breitbach C. J. Rose S. Bloomston M. (2013). Randomized dose-finding clinical trial of oncolytic immunotherapeutic vaccinia JX-594 in liver cancer. *Nat. Med.* 19 329–336. 10.1038/nm.3089 23396206 PMC4268543

[B54] Hewitt S. L. Bai A. Bailey D. Ichikawa K. Zielinski J. Karp R. (2019). Durable anticancer immunity from intratumoral administration of IL-23, IL-36γ, and OX40L mRNAs. *Sci. Transl. Med.* 11:aat9143. 10.1126/scitranslmed.aat9143 30700577

[B55] Hirvinen M. Rajecki M. Kapanen M. Parviainen S. Rouvinen-Lagerström N. Diaconu I. (2015). Immunological effects of a tumor necrosis factor alpha-armed oncolytic adenovirus. *Hum. Gene Ther.* 26 134–144. 10.1089/hum.2014.069 25557131

[B56] Hodi F. S. O’Day S. J. McDermott D. F. Weber R. W. Sosman J. A. Haanen J. B. (2010). Improved survival with ipilimumab in patients with metastatic melanoma. *N. Engl. J. Med.* 363 711–723. 10.1056/NEJMoa1003466 20525992 PMC3549297

[B57] Hong I.-S. (2016). Stimulatory versus suppressive effects of GM-CSF on tumor progression in multiple cancer types. *Exp. Mol. Med.* 48:e242. 10.1038/emm.2016.64 27364892 PMC4973317

[B58] Huang P. Kaku H. Chen J. Kashiwakura Y. Saika T. Nasu Y. (2010). Potent antitumor effects of combined therapy with a telomerase-specific, replication-competent adenovirus (OBP-301) and IL-2 in a mouse model of renal cell carcinoma. *Cancer Gene Ther.* 17 484–491. 10.1038/cgt.2010.5 20168351

[B59] Huang P. Watanabe M. Kaku H. Kashiwakura Y. Chen J. Saika T. (2008). Direct and distant antitumor effects of a telomerase-selective oncolytic adenoviral agent, OBP-301, in a mouse prostate cancer model. *Cancer Gene Ther.* 15 315–322. 10.1038/cgt.2008.3 18274558

[B60] Ismail A. M. Zhou X. Dyer D. W. Seto D. Rajaiya J. Chodosh J. (2019). Genomic foundations of evolution and ocular pathogenesis in human adenovirus species D. *FEBS Lett.* 593 3583–3608. 10.1002/1873-3468.13693 31769017 PMC7185199

[B61] Jain R. K. Stylianopoulos T. (2010). Delivering nanomedicine to solid tumors. *Nat. Rev. Clin. Oncol.* 7 653–664. 10.1038/nrclinonc.2010.139 20838415 PMC3065247

[B62] Jiang H. Rivera-Molina Y. Gomez-Manzano C. Clise-Dwyer K. Bover L. Vence L. M. (2017). Oncolytic Adenovirus and Tumor-Targeting Immune Modulatory Therapy Improve Autologous Cancer Vaccination. *Cancer Res.* 77 3894–3907. 10.1158/0008-5472.CAN-17-0468 28566332 PMC5549681

[B63] Jiang H. Shin D. H. Nguyen T. T. Fueyo J. Fan X. Henry V. (2019). Localized Treatment with Oncolytic Adenovirus Delta-24-RGDOX Induces Systemic Immunity against Disseminated Subcutaneous and Intracranial Melanomas. *Clin. Cancer Res.* 25 6801–6814. 10.1158/1078-0432.Ccr-19-0405 31455679 PMC6858961

[B64] Jonas R. A. Ung L. Rajaiya J. Chodosh J. (2020). Mystery eye: Human adenovirus and the enigma of epidemic keratoconjunctivitis. *Prog. Retin. Eye Res.* 76:100826. 10.1016/j.preteyeres.2019.100826 31891773 PMC7302964

[B65] Kanaya N. Kuroda S. Kakiuchi Y. Kumon K. Tsumura T. Hashimoto M. (2020). Immune Modulation by Telomerase-Specific Oncolytic Adenovirus Synergistically Enhances Antitumor Efficacy with Anti-PD1 Antibody. *Mol. Ther.* 28 794–804. 10.1016/j.ymthe.2020.01.003 31991110 PMC7054725

[B66] Kanerva A. Nokisalmi P. Diaconu I. Koski A. Cerullo V. Liikanen I. (2013). Antiviral and antitumor T-cell immunity in patients treated with GM-CSF-coding oncolytic adenovirus. *Clin. Cancer Res.* 19 2734–2744. 10.1158/1078-0432.CCR-12-2546 23493351

[B67] Kennedy L. B. Salama A. K. S. (2020). A review of cancer immunotherapy toxicity. *CA Cancer J. Clin.* 70:21596. 10.3322/caac.21596 31944278

[B68] Kent L. N. Leone G. (2019). The broken cycle: E2F dysfunction in cancer. *Nat. Rev. Cancer* 19 326–338. 10.1038/s41568-019-0143-7 31053804

[B69] Kim J. Lee B. Kim J. S. Yun C. O. Kim J. H. Lee Y. J. (2002). Antitumoral effects of recombinant adenovirus YKL-1001, conditionally replicating in alpha-fetoprotein-producing human liver cancer cells. *Cancer Lett.* 180 23–32. 10.1016/s0304-3835(02)00017-4 11911966

[B70] Kitajima S. Li F. Takahashi C. (2020). Tumor Milieu Controlled by RB Tumor Suppressor. *Int. J. Mol. Sci.* 21:ijms21072450. 10.3390/ijms21072450 32244804 PMC7177274

[B71] Kobayashi Y. Lim S.-O. Yamaguchi H. (2020). Oncogenic signaling pathways associated with immune evasion and resistance to immune checkpoint inhibitors in cancer. *Semin. Cancer Biol.* 65 51–64. 10.1016/j.semcancer.2019.11.011 31874279

[B72] Komita H. Zhao X. Katakam A. K. Kumar P. Kawabe M. Okada H. (2009). Conditional interleukin-12 gene therapy promotes safe and effective antitumor immunity. *Cancer Gene Ther.* 16 883–891. 10.1038/cgt.2009.33 19444303 PMC3427922

[B73] Koodie L. Robertson M. G. Chandrashekar M. Ruth G. Dunning M. Bianco R. W. (2019). Rodents Versus Pig Model for Assessing the Performance of Serotype Chimeric Ad5/3 Oncolytic Adenoviruses. *Cancers* 11:cancers11020198. 10.3390/cancers11020198 30744019 PMC6406826

[B74] Korniluk A. Kemona H. Dymicka-Piekarska V. (2014). Multifunctional CD40L: pro- and anti-neoplastic activity. *Tumour Biol.* 35 9447–9457. 10.1007/s13277-014-2407-x 25117071 PMC4213374

[B75] Kosti P. Maher J. Arnold J. N. (2018). Perspectives on Chimeric Antigen Receptor T-Cell Immunotherapy for Solid Tumors. *Front. Immunol.* 9:1104. 10.3389/fimmu.2018.01104 29872437 PMC5972325

[B76] Kowanetz M. Wu X. Lee J. Tan M. Hagenbeek T. Qu X. (2010). Granulocyte-colony stimulating factor promotes lung metastasis through mobilization of Ly6G+Ly6C+ granulocytes. *Proc. Natl. Acad. Sci. U S A.* 107 21248–21255. 10.1073/pnas.1015855107 21081700 PMC3003076

[B77] Kuryk L. Møller A.-S. W. Jaderberg M. (2019). Combination of immunogenic oncolytic adenovirus ONCOS-102 with anti-PD-1 pembrolizumab exhibits synergistic antitumor effect in humanized A2058 melanoma huNOG mouse model. *Oncoimmunology* 8:e1532763. 10.1080/2162402X.2018.1532763 30713786 PMC6343802

[B78] Lang F. F. Conrad C. Gomez-Manzano C. Yung W. K. A. Sawaya R. Weinberg J. S. (2018). Phase I Study of DNX-2401 (Delta-24-RGD) Oncolytic Adenovirus: Replication and Immunotherapeutic Effects in Recurrent Malignant Glioma. *J. Clin. Oncol.* 36 1419–1427. 10.1200/JCO.2017.75.8219 29432077 PMC6075856

[B79] Lee M. Lu Z. H. Li J. Kashentseva E. A. Dmitriev I. P. Mendonca S. A. (2020). Targeting Tumor Neoangiogenesis via Targeted Adenoviral Vector to Achieve Effective Cancer Gene Therapy for Disseminated Neoplastic Disease. *Mol. Cancer Ther.* 19 966–971. 10.1158/1535-7163.MCT-19-0768 31907220 PMC7155772

[B80] Leonard J. P. Sherman M. L. Fisher G. L. Buchanan L. J. Larsen G. Atkins M. B. (1997). Effects of single-dose interleukin-12 exposure on interleukin-12-associated toxicity and interferon-gamma production. *Blood* 90 2541–2548. 9326219

[B81] Li B. Chan H. L. Chen P. (2019). Immune Checkpoint Inhibitors: Basics and Challenges. *Curr. Med. Chem.* 26 3009–3025. 10.2174/0929867324666170804143706 28782469

[B82] Lion T. (2014). Adenovirus infections in immunocompetent and immunocompromised patients. *Clin. Microbiol. Rev.* 27 441–462. 10.1128/CMR.00116-13 24982316 PMC4135893

[B83] Liu T.-C. Hallden G. Wang Y. Brooks G. Francis J. Lemoine N. (2004). An E1B-19 kDa gene deletion mutant adenovirus demonstrates tumor necrosis factor-enhanced cancer selectivity and enhanced oncolytic potency. *Mol. Ther.* 9 786–803. 10.1016/j.ymthe.2004.03.017 15194046

[B84] Lopez-Gordo E. Doszpoly A. Duffy M. R. Coughlan L. Bradshaw A. C. White K. M. (2017). Defining a Novel Role for the Coxsackievirus and Adenovirus Receptor in Human Adenovirus Serotype 5 Transduction in the Presence of Mouse Serum. *J. Virol.* 91:16. 10.1128/JVI.02487-16 28381574 PMC5446653

[B85] Lu C. S. Hsieh J. L. Lin C. Y. Tsai H. W. Su B. H. Shieh G. S. (2015). Potent antitumor activity of Oct4 and hypoxia dual-regulated oncolytic adenovirus against bladder cancer. *Gene Ther.* 22 305–315. 10.1038/gt.2014.122 25588741

[B86] Lubaroff D. M. Konety B. R. Link B. Gerstbrein J. Madsen T. Shannon M. (2009). Phase I clinical trial of an adenovirus/prostate-specific antigen vaccine for prostate cancer: safety and immunologic results. *Clin. Cancer Res.* 15 7375–7380. 10.1158/1078-0432.CCR-09-1910 19920098 PMC2787649

[B87] Lubaroff D. M. Williams R. D. Vaena D. Joudi F. Brown J. Smith M. (2012). An ongoing Phase II trial of an adenovirus/PSA vaccine for prostate cancer. *Cancer Res.* 72:1. 10.1158/1538-7445.Am2012-2692

[B88] Ma S. Li X. Wang X. Cheng L. Li Z. Zhang C. (2019). Current Progress in CAR-T Cell Therapy for Solid Tumors. *Int. J. Biol. Sci.* 15 2548–2560. 10.7150/ijbs.34213 31754328 PMC6854376

[B89] Machiels J.-P. Salazar R. Rottey S. Duran I. Dirix L. Geboes K. (2019). A phase 1 dose escalation study of the oncolytic adenovirus enadenotucirev, administered intravenously to patients with epithelial solid tumors (EVOLVE). *J. Immunother. Cancer* 7:20. 10.1186/s40425-019-0510-7 30691536 PMC6348630

[B90] Machitani M. Sakurai F. Wakabayashi K. Tomita K. Tachibana M. Mizuguchi H. (2016). Dicer functions as an antiviral system against human adenoviruses via cleavage of adenovirus-encoded noncoding RNA. *Sci. Rep.* 6:27598. 10.1038/srep27598 27273616 PMC4895142

[B91] Maeng H. Terabe M. Berzofsky J. A. (2018). Cancer vaccines: translation from mice to human clinical trials. *Curr. Opin. Immunol.* 51 111–122. 10.1016/j.coi.2018.03.001 29554495 PMC5943163

[B92] Mahalingam D. Wilkinson G. A. Eng K. H. Fields P. Raber P. Moseley J. L. (2020). Pembrolizumab in Combination with the Oncolytic Virus Pelareorep and Chemotherapy in Patients with Advanced Pancreatic Adenocarcinoma: A Phase Ib Study. *Clin. Cancer Res.* 26 71–81. 10.1158/1078-0432.CCR-19-2078 31694832 PMC6942612

[B93] Majhen D. Calderon H. Chandra N. Fajardo C. A. Rajan A. Alemany R. (2014). Adenovirus-based vaccines for fighting infectious diseases and cancer: progress in the field. *Hum. Gene Ther.* 25 301–317. 10.1089/hum.2013.235 24580050

[B94] Marshall A. E. Roes M. V. Passos D. T. DeWeerd M. C. Chaikovsky A. C. Sage J. (2019). Deletion in Retinoblastoma Protein Pathway-Disrupted Cells Results in DNA Damage and Cancer Progression. *Mol. Cell Biol.* 39:MCB.105–MCB.119. 10.1128/MCB.00105-19 31138663 PMC6664603

[B95] Martínez-Vélez N. Garcia-Moure M. Marigil M. González-Huarriz M. Puigdelloses M. Gallego Pérez-Larraya J. (2019). The oncolytic virus Delta-24-RGD elicits an antitumor effect in pediatric glioma and DIPG mouse models. *Nat. Commun.* 10:2235. 10.1038/s41467-019-10043-0 31138805 PMC6538754

[B96] Masoud S. J. Hu J. B. Beasley G. M. Stewart J. H. Mosca P. J. (2019). Efficacy of Talimogene Laherparepvec (T-VEC) Therapy in Patients with In-Transit Melanoma Metastasis Decreases with Increasing Lesion Size. *Ann. Surg. Oncol.* 26 4633–4641. 10.1245/s10434-019-07691-3 31414290

[B97] McConnell M. J. Imperiale M. J. (2004). Biology of adenovirus and its use as a vector for gene therapy. *Hum. Gene Ther.* 15 1022–1033. 10.1089/hum.2004.15.1022 15610603

[B98] Miksad R. A. Meropol N. J. (2018). Carcinoembryonic Antigen-Still More to Learn From the Real World. *JAMA Oncol.* 4 315–316. 10.1001/jamaoncol.2017.4408 29270625

[B99] Moradi A. Srinivasan S. Clements J. Batra J. (2019). Beyond the biomarker role: prostate-specific antigen (PSA) in the prostate cancer microenvironment. *Cancer Metastasis Rev.* 38 333–346. 10.1007/s10555-019-09815-3 31659564

[B100] Morse M. A. Chaudhry A. Gabitzsch E. S. Hobeika A. C. Osada T. Clay T. M. (2013). Novel adenoviral vector induces T-cell responses despite anti-adenoviral neutralizing antibodies in colorectal cancer patients. *Cancer Immunol. Immunother.* 62 1293–1301. 10.1007/s00262-013-1400-3 23624851 PMC3732790

[B101] Nguyen K. G. Vrabel M. R. Mantooth S. M. Hopkins J. J. Wagner E. S. Gabaldon T. A. (2020). Localized Interleukin-12 for Cancer Immunotherapy. *Front. Immunol.* 11:575597. 10.3389/fimmu.2020.575597 33178203 PMC7593768

[B102] Niemann J. Woller N. Brooks J. Fleischmann-Mundt B. Martin N. T. Kloos A. (2019). Molecular retargeting of antibodies converts immune defense against oncolytic viruses into cancer immunotherapy. *Nat. Commun.* 10:3236. 10.1038/s41467-019-11137-5 31324774 PMC6642145

[B103] O’Donnell J. S. Teng M. W. L. Smyth M. J. (2019). Cancer immunoediting and resistance to T cell-based immunotherapy. *Nat. Rev. Clin. Oncol.* 16 151–167. 10.1038/s41571-018-0142-8 30523282

[B104] Oh E. Hong J. Kwon O.-J. Yun C.-O. (2018). A hypoxia- and telomerase-responsive oncolytic adenovirus expressing secretable trimeric TRAIL triggers tumour-specific apoptosis and promotes viral dispersion in TRAIL-resistant glioblastoma. *Sci. Rep.* 8:1420. 10.1038/s41598-018-19300-6 29362367 PMC5780382

[B105] Packiam V. T. Lamm D. L. Barocas D. A. Trainer A. Fand B. Davis R. L. (2018). An open label, single-arm, phase II multicenter study of the safety and efficacy of CG0070 oncolytic vector regimen in patients with BCG-unresponsive non-muscle-invasive bladder cancer: Interim results. *Urol. Oncol.* 36 440–447. 10.1016/j.urolonc.2017.07.005 28755959

[B106] Pardoll D. M. (2012). The blockade of immune checkpoints in cancer immunotherapy. *Nat. Rev. Cancer* 12 252–264. 10.1038/nrc3239 22437870 PMC4856023

[B107] Pascual-Pasto G. Bazan-Peregrino M. Olaciregui N. G. Restrepo-Perdomo C. A. Mato-Berciano A. Ottaviani D. (2019). Therapeutic targeting of the RB1 pathway in retinoblastoma with the oncolytic adenovirus VCN-01. *Sci. Transl. Med.* 11:aat9321. 10.1126/scitranslmed.aat9321 30674657

[B108] Pesonen S. Diaconu I. Cerullo V. Escutenaire S. Raki M. Kangasniemi L. (2012). Integrin targeted oncolytic adenoviruses Ad5-D24-RGD and Ad5-RGD-D24-GMCSF for treatment of patients with advanced chemotherapy refractory solid tumors. *Int. J. Cancer* 130 1937–1947. 10.1002/ijc.26216 21630267

[B109] Pol J. G. Acuna S. A. Yadollahi B. Tang N. Stephenson K. B. Atherton M. J. (2019). Preclinical evaluation of a MAGE-A3 vaccination utilizing the oncolytic Maraba virus currently in first-in-human trials. *Oncoimmunology* 8:e1512329. 10.1080/2162402X.2018.1512329 30546947 PMC6287790

[B110] Pol J. G. Atherton M. J. Bridle B. W. Stephenson K. B. Le Boeuf F. Hummel J. L. (2018). Development and applications of oncolytic Maraba virus vaccines. *Oncolyt. Virother.* 7 117–128. 10.2147/OV.S154494 30538968 PMC6263248

[B111] Porter C. E. Rosewell Shaw A. Jung Y. Yip T. Castro P. D. Sandulache V. C. (2020). Oncolytic Adenovirus Armed with BiTE, Cytokine, and Checkpoint Inhibitor Enables CAR T Cells to Control the Growth of Heterogeneous Tumors. *Mol. Ther.* 28 1251–1262. 10.1016/j.ymthe.2020.02.016 32145203 PMC7210703

[B112] Prassas I. Eissa A. Poda G. Diamandis E. P. (2015). Unleashing the therapeutic potential of human kallikrein-related serine proteases. *Nat. Rev. Drug Discov.* 14 183–202. 10.1038/nrd4534 25698643

[B113] Radke J. R. Cook J. L. (2018). Human adenovirus infections: update and consideration of mechanisms of viral persistence. *Curr. Opin. Infect. Dis.* 31 251–256. 10.1097/QCO.0000000000000451 29601326 PMC6367924

[B114] Ramesh N. Ge Y. Ennist D. L. Zhu M. Mina M. Ganesh S. (2006). CG0070, a conditionally replicating granulocyte macrophage colony-stimulating factor–armed oncolytic adenovirus for the treatment of bladder cancer. *Clin. Cancer Res.* 12 305–313. 10.1158/1078-0432.ccr-05-1059 16397056

[B115] Ranki T. Pesonen S. Hemminki A. Partanen K. Kairemo K. Alanko T. (2016). Phase I study with ONCOS-102 for the treatment of solid tumors - an evaluation of clinical response and exploratory analyses of immune markers. *J. Immunother. Cancer* 4:17. 10.1186/s40425-016-0121-5 26981247 PMC4791966

[B116] Robert-Guroff M. (2007). Replicating and non-replicating viral vectors for vaccine development. *Curr. Opin. Biotechnol.* 18 546–556. 10.1016/j.copbio.2007.10.010 18063357 PMC2245896

[B117] Rosenberg S. A. (2014). IL-2: the first effective immunotherapy for human cancer. *J. Immunol.* 192 5451–5458. 10.4049/jimmunol.1490019 24907378 PMC6293462

[B118] Rosewell Shaw A. Porter C. E. Watanabe N. Tanoue K. Sikora A. Gottschalk S. (2017). Adenovirotherapy Delivering Cytokine and Checkpoint Inhibitor Augments CAR T Cells against Metastatic Head and Neck Cancer. *Mol. Ther.* 25 2440–2451. 10.1016/j.ymthe.2017.09.010 28974431 PMC5675597

[B119] Roy S. Shirley P. S. McClelland A. Kaleko M. (1998). Circumvention of immunity to the adenovirus major coat protein hexon. *J. Virol.* 72 6875–6879. 10.1128/jvi.72.8.6875-6879.1998 9658137 PMC109897

[B120] Samson A. Scott K. J. Taggart D. West E. J. Wilson E. Nuovo G. J. (2018). Intravenous delivery of oncolytic reovirus to brain tumor patients immunologically primes for subsequent checkpoint blockade. *Sci. Transl. Med.* 10:aam7577. 10.1126/scitranslmed.aam7577 29298869 PMC6276984

[B121] Santos J. M. Cervera-Carrascon V. Havunen R. Zafar S. Siurala M. Sorsa S. (2018). Adenovirus Coding for Interleukin-2 and Tumor Necrosis Factor Alpha Replaces Lymphodepleting Chemotherapy in Adoptive T Cell Therapy. *Mol. Ther.* 26 2243–2254. 10.1016/j.ymthe.2018.06.001 30017877 PMC6127851

[B122] Santos J. M. Heiniö C. Cervera-Carrascon V. Quixabeira D. C. A. Siurala M. Havunen R. (2020). Oncolytic adenovirus shapes the ovarian tumor microenvironment for potent tumor-infiltrating lymphocyte tumor reactivity. *J. Immunother. Cancer* 8:000188. 10.1136/jitc-2019-000188 31940588 PMC7057530

[B123] Sarvizadeh M. Ghasemi F. Tavakoli F. Sadat Khatami S. Razi E. Sharifi H. (2019). Vaccines for colorectal cancer: an update. *J. Cell Biochem.* 120 8815–8828. 10.1002/jcb.28179 30536960

[B124] Sharma P. Allison J. P. (2015). The future of immune checkpoint therapy. *Science* 348 56–61. 10.1126/science.aaa8172 25838373

[B125] Shehata M. Schwarzmeier J. D. Nguyen S. T. Hilgarth M. Berger R. Hubmann R. (2000). Reconstitution of endogenous interferon a by recombinant interferon in hairy cell leukemia. *Cancer Res.* 60 5420–5426. 11034083

[B126] Sim G. C. Radvanyi L. (2014). The IL-2 cytokine family in cancer immunotherapy. *Cytokine Growth Factor Rev.* 25 377–390. 10.1016/j.cytogfr.2014.07.018 25200249

[B127] Spranger S. Bao R. Gajewski T. F. (2015). Melanoma-intrinsic β-catenin signalling prevents anti-tumour immunity. *Nature* 523 231–235. 10.1038/nature14404 25970248

[B128] Sung H. Ferlay J. Siegel R. L. Laversanne M. Soerjomataram I. Jemal A. (2021). Global cancer statistics 2020: GLOBOCAN estimates of incidence and mortality worldwide for 36 cancers in 185 countries. *CA Cancer J. Clin.* 2021:21660. 10.3322/caac.21660 33538338

[B129] Taipale K. Liikanen I. Koski A. Heiskanen R. Kanerva A. Hemminki O. (2016). Predictive and Prognostic Clinical Variables in Cancer Patients Treated With Adenoviral Oncolytic Immunotherapy. *Mol. Ther.* 24 1323–1332. 10.1038/mt.2016.67 27039846 PMC5088758

[B130] Tanoue K. Rosewell Shaw A. Watanabe N. Porter C. Rana B. Gottschalk S. (2017). Armed Oncolytic Adenovirus-Expressing PD-L1 Mini-Body Enhances Antitumor Effects of Chimeric Antigen Receptor T Cells in Solid Tumors. *Cancer Res.* 77 2040–2051. 10.1158/0008-5472.CAN-16-1577 28235763 PMC5392365

[B131] Tian X. Shen H. Li Z. Wang T. Wang S. (2019). Tumor-derived exosomes, myeloid-derived suppressor cells, and tumor microenvironment. *J. Hematol. Oncol.* 12:84. 10.1186/s13045-019-0772-z 31438991 PMC6704713

[B132] Todaro M. Gaggianesi M. Catalano V. Benfante A. Iovino F. Biffoni M. (2014). CD44v6 is a marker of constitutive and reprogrammed cancer stem cells driving colon cancer metastasis. *Cell Stem Cell* 14 342–356. 10.1016/j.stem.2014.01.009 24607406

[B133] Tran T. Blanc C. Granier C. Saldmann A. Tanchot C. Tartour E. (2019). Therapeutic cancer vaccine: building the future from lessons of the past. *Semin. Immunopathol.* 41 69–85. 10.1007/s00281-018-0691-z 29978248

[B134] Tuve S. Liu Y. Tragoolpua K. Jacobs J. D. Yumul R. C. Li Z.-Y. (2009). In situ adenovirus vaccination engages T effector cells against cancer. *Vaccine* 27 4225–4239. 10.1016/j.vaccine.2009.03.074 19481312 PMC2727281

[B135] Vera B. Martínez-Vélez N. Xipell E. Acanda de la Rocha A. Patiño-García A. Saez-Castresana J. (2016). Characterization of the Antiglioma Effect of the Oncolytic Adenovirus VCN-01. *PLoS One* 11:e0147211. 10.1371/journal.pone.0147211 26808201 PMC4726573

[B136] Wang B. Liu J. Ma L. N. Xiao H. L. Wang Y. Z. Li Y. (2013). Chimeric 5/35 adenovirus-mediated Dickkopf-1 overexpression suppressed tumorigenicity of CD44^+^ gastric cancer cells via attenuating Wnt signaling. *J. Gastroenterol.* 48 798–808. 10.1007/s00535-012-0711-z 23188090

[B137] Wang C.-H. K. Chan L. W. Johnson R. N. Chu D. S. H. Shi J. Schellinger J. G. (2011). The transduction of Coxsackie and Adenovirus Receptor-negative cells and protection against neutralizing antibodies by HPMA-co-oligolysine copolymer-coated adenovirus. *Biomaterials* 32 9536–9545. 10.1016/j.biomaterials.2011.08.069 21959008 PMC3190026

[B138] Wang H. Li Z.-Y. Liu Y. Persson J. Beyer I. Möller T. (2011). Desmoglein 2 is a receptor for adenovirus serotypes 3, 7, 11 and 14. *Nat. Med.* 17:2270. 10.1038/nm.2270 21151137 PMC3074512

[B139] Wang P. Li X. Wang J. Gao D. Li Y. Li H. (2017). Re-designing Interleukin-12 to enhance its safety and potential as an anti-tumor immunotherapeutic agent. *Nat. Commun.* 8:1395. 10.1038/s41467-017-01385-8 29123084 PMC5680234

[B140] Wang X. Su C. Cao H. Li K. Chen J. Jiang L. (2008). A novel triple-regulated oncolytic adenovirus carrying p53 gene exerts potent antitumor efficacy on common human solid cancers. *Mol. Cancer Ther.* 7 1598–1603. 10.1158/1535-7163.MCT-07-2429 18566230

[B141] Wang Y. Hallden G. Hill R. Anand A. Liu T.-C. Francis J. (2003). E3 gene manipulations affect oncolytic adenovirus activity in immunocompetent tumor models. *Nat. Biotechnol.* 21 1328–1335. 10.1038/nbt887 14555956

[B142] Wang Z. Wu Z. Liu Y. Han W. (2017). New development in CAR-T cell therapy. *J. Hematol. Oncol.* 10:53. 10.1186/s13045-017-0423-1 28222796 PMC5320663

[B143] Webb G. J. Hirschfield G. M. Lane P. J. L. (2016). OX40, OX40L and Autoimmunity: a Comprehensive Review. *Clin. Rev. Allergy Immunol.* 50 312–332. 10.1007/s12016-015-8498-3 26215166

[B144] Wenthe J. Naseri S. Hellström A.-C. Wiklund H. J. Eriksson E. Loskog A. (2020). Immunostimulatory oncolytic virotherapy for multiple myeloma targeting 4-1BB and/or CD40. *Cancer Gene Ther.* 2020 176–179. 10.1038/s41417-020-0176-9 32355275 PMC7725669

[B145] Wherry E. J. Kurachi M. (2015). Molecular and cellular insights into T cell exhaustion. *Nat. Rev. Immunol.* 15 486–499. 10.1038/nri3862 26205583 PMC4889009

[B146] Xia L. Liu Y. Wang Y. (2019). PD-1/PD-L1 Blockade Therapy in Advanced Non-Small-Cell Lung Cancer: Current Status and Future Directions. *Oncologist* 24(Suppl. 1), S31–S41. 10.1634/theoncologist.2019-IO-S1-s05 30819829 PMC6394772

[B147] Xia T. Konno H. Barber G. N. (2016). Recurrent Loss of STING Signaling in Melanoma Correlates with Susceptibility to Viral Oncolysis. *Cancer Res.* 76 6747–6759. 10.1158/0008-5472.CAN-16-1404 27680683

[B148] Yamamoto Y. Nagasato M. Yoshida T. Aoki K. (2017). Recent advances in genetic modification of adenovirus vectors for cancer treatment. *Cancer Sci.* 108 831–837. 10.1111/cas.13228 28266780 PMC5448613

[B149] Yu B. Zhou Y. Wu H. Wang Z. Zhan Y. Feng X. (2012). Seroprevalence of neutralizing antibodies to human adenovirus type 5 in healthy adults in China. *J. Med. Virol.* 84 1408–1414. 10.1002/jmv.23325 22825819

[B150] Yumul R. Richter M. Lu Z.-Z. Saydaminova K. Wang H. Wang C.-H. K. (2016). Epithelial Junction Opener Improves Oncolytic Adenovirus Therapy in Mouse Tumor Models. *Hum. Gene Ther.* 27 325–337. 10.1089/hum.2016.022 26993072 PMC4840918

[B151] Zafar S. Basnet S. Launonen I.-M. Quixabeira D. C. A. Santos J. Hemminki O. (2020a). Oncolytic adenovirus type 3 coding for CD40L facilitates dendritic cell therapy of prostate cancer in humanized mice and patient samples. *Hum. Gene Ther.* 2020:2020.222. 10.1089/hum.2020.222 33050725 PMC10112462

[B152] Zafar S. Quixabeira D. C. A. Kudling T. V. Cervera-Carrascon V. Santos J. M. Grönberg-Vähä-Koskela S. (2020b). Ad5/3 is able to avoid neutralization by binding to erythrocytes and lymphocytes. *Cancer Gene Ther.* 2020:00226–z. 10.1038/s41417-020-00226-z 32920593 PMC8119244

[B153] Zaremba S. Barzaga E. Zhu M. Soares N. Tsang K. Y. Schlom J. (1997). Identification of an enhancer agonist cytotoxic T lymphocyte peptide from human carcinoembryonic antigen. *Cancer Res.* 57 4570–4577. 9377571

[B154] Zhao Z. Zheng L. Chen W. Weng W. Song J. Ji J. (2019). Delivery strategies of cancer immunotherapy: recent advances and future perspectives. *J. Hematol. Oncol.* 12:126. 10.1186/s13045-019-0817-3 31779642 PMC6883629

